# Optimization of a high-throughput nanofluidic real-time PCR to detect and quantify of 15 bacterial species and 92 Streptococcus pneumoniae serotypes

**DOI:** 10.1038/s41598-023-31820-4

**Published:** 2023-03-21

**Authors:** Sarah L. Downs, Shabir. A. Madhi, Lara van der Merwe, Marta. C. Nunes, Courtney P. Olwagen

**Affiliations:** 1 South African Medical Research Council, Vaccines and Infectious Diseases Analytics Research Unit, School of Pathology, Faculty of Health Sciences, University of the Witwatersrand, Johannesburg, South Africa; 2 Department of Science and Technology/National Research Foundation, South African Research Chair Initiative in Vaccine Preventable Diseases, Faculty of Health Sciences, University of the Witwatersrand, Johannesburg, South Africa

**Keywords:** High-throughput screening, Microbiology techniques, Nanobiotechnology, Medical research, Infectious diseases, Respiratory tract diseases, Nanobiotechnology

## Abstract

Sensitive tools for detecting concurrent colonizing pneumococcal serotypes are needed for detailed evaluation of the direct and indirect impact of routine pneumococcal conjugate vaccine (PCV) immunization. A high-throughput quantitative nanofluidic real-time PCR (Standard BioTools ‘Fluidigm’) reaction-set was developed to detect and quantify 92 pneumococcal serotypes in archived clinical samples. Nasopharyngeal swabs collected in 2009–2011 from South African children ≤ 5 years-old, previously serotyped with standard culture-based methods were used for comparison. The reaction-set within the ‘Fluidigm’ effectively amplified all targets with high efficiency (90–110%), reproducibility (R^2^ ≥ 0.98), and at low limit-of-detection (< 10^2^ CFU/ml). A blind analysis of 1 973 nasopharyngeal swab samples showed diagnostic sensitivity > 80% and specificity > 95% compared with the referent standard, culture based Quellung method. The qPCR method was able to serotype pneumococcal types with good discrimination compared with Quellung (ROC-AUC: > 0.73). The high-throughput nanofluidic real-time PCR method simultaneously detects 57 individual serotypes, and 35 serotypes within 16 serogroups in 96 samples (including controls), within a single qPCR run. This method can be used to evaluate the impact of current PCV formulations on vaccine-serotype and non-vaccine-serotype colonization, including detection of multiple concurrently colonizing serotypes. Our qPCR method can allow for monitoring of serotype-specific bacterial load, as well as emergence or ongoing transmission of minor or co-colonizing serotypes that may have invasive disease potential.

## Introduction

The capsule of *Streptococcus pneumoniae* is composed of repeating polysaccharides genetically encoded by the Capsular Polysaccharide Synthesis (CPS) locus that confers serotype heterogeneity^1,2^. There are 100 biochemically and serologically distinct *S. pneumoniae* serotypes^3^. Capsular diversity is an important virulence factor in *S. pneumoniae*^4^ and serotypes have different potential to cause disease in humans^5^. Pneumococcal Conjugate Vaccines (PCV) prevent acquisition of pneumococcal vaccine serotype (VT) carriage, which is a precursor to disease^6–10^. The first licensed PCV (PCV7) targeted seven serotypes, namely: 4, 6B, 9V, 14, 18C, 19F, and 23F. Current PCVs in use in our setting include an additional three (PCV10: 1, 5 and 7F) and six (PCV13: 1, 3, 5, 6A, 7F and 19A) serotypes. Next generation 15- and 20-valent PCVs are under clinical trials and include an additional two (22F and 33F)^11^ and seven (8, 10A, 11A, 12F, 15B, 22F and 33F)^12^ serotypes, respectively, compared with PCV13.

Surveillance of pneumococci colonization of the upper airway is useful to assess the impact of PCV immunization at a population level^13^. Pneumococcal colonization surveys are best performed using comprehensive, sensitive, and accurate serotyping methods able to detect multiple serotype co-carriage, including VT and non-vaccine serotypes (NVT). Consistent surveillance of circulating serotypes is needed to inform vaccination strategies and utilization of higher or alternate valency PCVs, including tailored to different geographic regions; and for the detection of emerging or replacement serotypes^13^.

Culture-based Quellung methods remain the gold standard for *S. pneumoniae* serotyping; however, these methods are time-consuming, with low sensitivity, and are costly^14,15^. Compared with culture-based methods, Polymerase Chain Reaction (PCR)-based detection and serotyping do not rely on organism viability that can be negatively affected by antibiotic use, sample transport and storage conditions, are less time consuming, provide faster diagnosis and higher sensitivity^15^. Conventional and real-time PCR have been used, including single tube nested and multiplex PCRs with gel or capillary electrophoresis and combined with dot-blot detection, culture enrichment and sequencing-based analysis^16–28^. Real-time PCR methods have been developed with an increasing number of serotypes detected^21,29–31^, including in high-throughput systems^15,32,33^. Further, quantitative PCR (qPCR) methods that are validated to determine bacterial load^29,31,32,34^ are important for assessing the impact of vaccines in public health programs. Increased bacterial load in the nasopharynx may predict serotype-specific invasive disease potential^31^.

Previously we developed a nanofluidic qPCR reaction-set that was able to simultaneously detect and quantify 55 pneumococcal serotypes^32^, as well as a detection and typing method to distinguish serogroups 6A/B/C/D, 18A/B/C, and 22A/F into single serotypes^35^ using the same platform. Here we expand on our method to detect and quantify an additional 38 serotypes—allowing for comprehensive pneumococcal serotyping (92 serotypes) and detection of 15 other bacterial species to assess colonization and bacterial load in respiratory samples.

## Methods

### Reaction-set development

To develop a comprehensive reaction-set for serotyping *S. pneumoniae* and detecting other species that may be present in the nasopharynx, within an in-house, nanofluidic, high-throughput, real-time PCR platform, a review of published assay-set (primers and probes) sequences was conducted. Representative GenBank FASTA sequences for serotype 19B (CR931676) and 19F (CR931678) capsular genes, the pneumococcal Xisco putative protein gene (NC_003028) and the *Haemophilus influenzae* Bex encapsulation gene (X54987) were aligned in BioEdit using ClustalW Multiple alignment^36,37^. Standard primers and dye-labelled MGB probes were manually designed to detect the 19F capsular polysaccharide gene *wzh* to differentiate 19F from 19B^1,2^, to detect the pneumococcal Xisco gene and to detect the *H. influenzae* Bex*A* gene. All curated oligonucleotide sequences for each assay-set listed in Supplementary Table S1 were analyzed to ensure specificity *in-silico* using the Basic Local Alignment Search Tool (NCBI BLAST; http://www.ncbi.nlm.nih.gov/BLAST/) prior to inclusion in the reaction-set. The melting temperature (Tm) of individual oligonucleotides within each assay-set was determined using the online tools Oligocalc^38^ and the Integrated DNA Technologies (IDT) OligoAnalyzer (http://eu.idtdna.com/analyzer/Applications/OligoAnalyzer/) to assess their suitability for inclusion within a uniform thermal profile and multiplexing. The IDT OligoAnalyzer tool was used to assess secondary structures and self-binding sites. The analytical efficacy (efficiency, sensitivity, and specificity) and diagnostic performance (sensitivity, specificity, and concordance) of the concatenated reaction-set within the nanofluidic high-throughput real-time qPCR (‘Fluidigm’, Standard BioTools) was established through calibrators and clinical samples respectively.

### Bacterial strains

Control strains were grown according to standard microtiter culture methods to quantify their relative density as colony-forming units (CFU/ml) for use as quantification calibrators and to assess analytical efficacy. Total DNA was extracted and stored at − 30 °C until assayed, as described previously^35^. DNA from 89 isolates that were representative of the included pneumococcal serotypes and 15 other bacteria were used to optimize the PCR assay-sets and assess the analytical specificity of the assay-sets to their respective targets (pneumococcal serotypes: 1; 2; 3; 4; 5; 6A; 6B; 6C; 6D; 7A; 7B; 7F; 7C; 8; 9A; 9L; 9N; 9V; 10A; 10B; 10C; 11A; 11B; 11C; 11D; 11F; 12B; 12F; 13; 14; 15A; 15B; 15C; 15F; 16A; 16F; 17A; 17F; 18A; 18B; 18C; 18F; 19A; 19B; 19C; 19F; 20; 21; 22A; 22F; 23A; 23B; 23F; 24A; 24B; 24F; 25A; 25F; 27; 28A; 28F; 29; 31; 32A; 32F; 33A; 33B; 33C; 33D; 33F; 34; 35A; 35C; 35F; 35B; 36; 37; 38; 39; 40; 41A; 42; 43; 44; 45; 46; 47A; 47F; 48; *Acinetobacter baumannii*, *Bordetella holmesii, B. parapertussis, B. pertussis, Escherichia coli, Haemophilus influenzae-b*, *non-typeable Haemophilus influenzae*, *Klebsiella pneumoniae*, *Moraxella catarrhalis*, *Neisseria lactamica*, *Neisseria meningitidis*, *Staphylococcus aureus*, *Streptococcus algalactiae* and *Streptococcus pyogenes*). The control isolates were obtained from the National Institute for Communicable Diseases (NICD), South Africa, the Murdoch Children’s Research Institute (MCRI), Australia and the South African Medical Research Council Vaccines and Infectious Diseases Analytics Research Unit (Wits-VIDA), Soweto, South Africa.

### Synthetic external calibrators

We utilized synthetic double-stranded DNA (dsDNA) template gene fragments (gBlocks) as external calibrators (Supplementary Tables S2 and S3) for analytical efficacy and intra- and inter-assay variation. The gBlocks contained sequences for each pneumococcal serotype or other bacterial target according to the pool (Supplementary Table S4) in which the respective assay-sets were included. These were applied to assess the analytical sensitivity (Limit of Detection) and repeatability (intra- and inter-assay variation and Levy-Jennings plots) of the reaction-set. The design, properties and sequences of each gBlock have been detailed previously^35^. Briefly, for each of the three pools (A, B and C, Supplementary Table S4) three gBlocks were designed (nine in total) that each included nine to thirteen target regions. Target sequences within gBlocks are easily quantified as there is an equimolar ratio of all templates. The amount of gBlock synthetic DNA was measured using the Thermo Fisher Invitrogen Qubit 2.0 Fluorometer Qubit 2.0 with the Invitrogen Qubit dsDNA BR Assay Kit. The copy number, or the number of gene equivalents was calculated using the formula:

$$Number\, of\, Copies = \frac{{amount\left( {ng} \right)*6.022 \times 10^{23}\, molecules\, per\, mol}}{{length \left( {bp} \right)*660*1 \times 10^{9} }}$$

The amount was the average of the three Qubit measurements which was multiplied by the Avogadro’s number (6.022 × 10^23^). The length in base pairs was multiplied by the average weight of a dsDNA nucleotide (660 Daltons or g/mole) and converted back to ng (1 × 10^9^).

### Clinical samples (nasopharyngeal swabs) for validation

**Figure 1:**
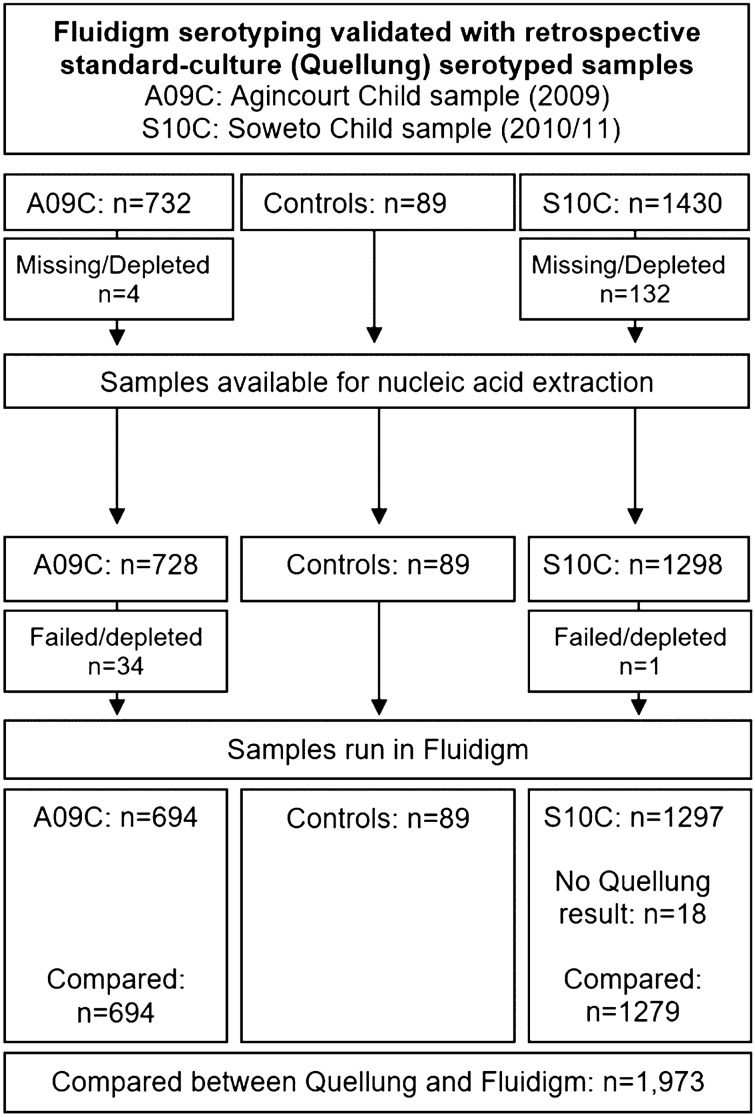
Validation flowchart describing the included archived clinical samples that were previously serotyped using the culture based Quellung method and that were compared with the Standard BioTools ‘Fluidigm’ real-time quantitative and serotyping PCR. A09C indicates samples collected from Agincourt (2009) children^39^. S10C indicates samples from Soweto (2010/11) children^40^. Samples were archived nasopharyngeal swabs in STGG as described in "Clinical samples (nasopharyngeal swabs) for validation" section, that were previously tested using the culture based Quellung method^39,40^. Archived clinical samples were used to assess the diagnostic performance of the ‘Fluidigm’ qPCR. Where samples were indicated as missing/depleted, these samples were not available for nucleic acid extraction as they were either exhausted through previous testing, or the sample vial was not located. Where samples are indicated as failed/depleted, the nucleic acid extraction failed and there was insufficient remaining sample to repeat this extraction. Control samples were culture strains used to assess the analytical performance of the ‘Fluidigm’ qPCR and are described in methods "Total nucleic acid extraction" section. All extracted samples and controls were run in the ‘Fluidigm’, however only samples with a Quellung result (positive or negative) were included in comparisons of diagnostic performance.

In May to October 2009 and May 2010 to February 2011, two carriage surveys were conducted in Agincourt, Mpumalanga, and Soweto, Gauteng respectively, to assess serotype-specific pneumococcal colonization during the early phase of introduction of PCV7 in South Africa which started in May 2009^39,40^. Carriage isolates from these cross-sectional surveys were previously cultured using standard microbiological methods and serotyped using the Quellung method. Here, we included all available archived nasopharyngeal swabs (NPS) from children aged 0 to 5-years-old to assess our method for pneumococcal serotyping in the age-groups where pneumococcal colonization was the highest (Fig. 1). Retrospectively, all archived samples, regardless of culture results were re-tested with ‘Fluidigm’, unless samples were depleted, or no Quellung results were available, to validate the reaction-set for accurate serotyping in clinical samples as outlined in Fig. 1. Out of the 1973 archived clinical samples available for the current analysis, 69.2% were positive for pneumococcus by culture.

### Total nucleic acid extraction

Samples (NPS in Skim-milk-tryptone-glucose-glycerin transport media [STGG]) that were stored at − 80 °C were thawed and vortexed at low speed for 30 s. Nucleic acids were extracted from 400 µl of STGG and eluted into 100 µl of elution buffer using the automated BioMérieux NucliSens easyMAG (BioMérieux, Marcy l′Etoile, France) nucleic acid extraction platform according to standard manufacturer protocols. One no-template-control (NTC) that was blank STGG was included in each batch of 23 clinical samples extracted. Nucleic acids were stored at − 30 °C until assayed.

### BioMark HD Nanofluidic qPCR loading, sample, and assay preparation

Specific target amplification (STA) was done as per manufactures recommendations as the initial step (pre-amplification of DNA) for the Biomark HD system (Standard BioTools, formerly Fluidigm) as discussed previously^35^. In short, assay-sets (primers only) were separated into three STA multiplex pools (Supplementary Table S3) to prevent any primers’ cross-reactivity. After STA, qPCR was carried out on either the Standard BioTools 96.96 Gene Expression (GE) Dynamic Array integrated fluidic circuit (IFC) (product number BMK-M-96.96) or the Flex Six 12.12 GE IFC (product number 100-6308) against a reaction-set that combines 96 samples with 96 assays-sets for 9 216 individual qPCR reactions per array or 12 samples with 12 assay-sets for 144 reactions per array, respectively. A flow diagram of the preparation and loading of the Standard BioTools (‘Fluidigm’) 96.96 IFC is detailed in Supplementary Fig. S1 and has been described in detail previously^35^. Briefly, for each sample, STA product from each of three pools (5 µL) and 10 µL of molecular grade H_2_O was combined, to dilute any remaining primers. Subsequently, assay premix for each target was aspirated into the IFC assay inlets for a final concentration of 9 µM primers and 2.5 µM probe per reaction and samples (2.25 µL Pooled STA product, 2.50 µL Applied Biosystems TaqMan Gene Expression Master Mix, catalog number 4370074 and 0.25 µL Standard BioTools/Fluidigm sample loading reagent) were aspirated into sample inlets. The IFC was then run in the BiomarkHD thermocycler, using the manufacturer supplied thermal cycling conditions: 50 °C for 2 min, 70 °C for 30 min, 25 °C for 10 min, 50 °C for 2 min, 96.5 °C for 10 min followed by 40 cycles of 96 °C for 15 s, 60 °C for 60 s. Results were analyzed using the Fluidigm Real-time PCR Analysis software. Here, thresholds were manually defined; the baseline was automatically assigned, and a Cycle of quantification (Cq) cut off value of 38 was applied.

### Analytical efficiency, sensitivity, specificity, and quantification of density

Regression calibration curves (standard curves) were prepared for each assay-set using duplicate tenfold serial dilutions of the targeted positive control strains or gBlocks. The standard curves included five data points; outliers at the end of the range that did not contain linear cycle of quantification (Cq) values were removed to define the linear dynamic range and determination coefficient (r^2^) value. At high template concentration, PCR inhibitors are at a higher concentration, whereas in highly diluted samples, stochastic binding results in variability in amplification. Concentrated or diluted template, or dilution errors will therefore affect the amplification efficiency, resulting in unpredictable Cq values. Values that fall outside of the limit of linearity, are no longer predicted by the linear equation. For this reason, outliers are excluded. The efficiency (%) for each included assay-set was derived from the slope (m) of the linear equation (y = mx + c) generated from the standard curves using the equation:

$$E = \left[ { - 1 + 10^{{\left( { - \frac{1}{m}} \right)}} } \right] \times 100$$

A triplicate dilution-series of bacterial control strains or gBlocks at the lower end of the linear dynamic range (10^3^–10^0^ copies per µL) were used to determine the analytical sensitivity or Limit of Detection (LOD) defined as lowest CFU/mL or copies detected in triplicate of ‘Fluidigm’ qPCR. A DNA library was prepared of the targeted pneumococcal serotypes or other bacterial species at an average 10^3^–10^4^ CFU/mL. The analytical specificity was assessed by running the DNA library against each assay-set in the ‘Fluidigm’ qPCR.

For assay-sets that were within the prescribed ranges for efficiency (90–110%), relative quantification of bacterial density was extrapolated using the linear equation generated from the standard curves of the calibrators (control strains and gBlocks of known density) using the equation:

$$Density = 10^{{\frac{Cq - c}{m} \times \frac{extraction\, volume}{{elution\, volume}}}}$$

Where more than one assay-set detected or determined a serotype, the average density was calculated. For example, to calculate the density of serotype 6A:

$$Density\, 6A = Density \left( {6ABCD + 6AC} \right)/2$$

The relative density between serotypes was used to assign hierarchy in multiple concurrent serotype carriage, with primary serotypes being those with the highest density, and sub-dominant or co-colonizing serotypes being those with lower relative density. Bland–Altman plots (difference vs. means) were constructed to compare the density of Lyt*A* to Pia*B*, and to compare the average pneumococcal density (Lyt*A* and Pia*B*) to the sum of serotype density per sample.

### Diagnostic sensitivity

The diagnostic sensitivity was assessed through blinded re-analysis of the stored clinical samples (n = 1973; Fig. 1) that were previously cultured and serotyped using the Quellung method. The samples were re-tested with the qPCR reaction-set within the 96.96 IFC that included the extracted NTC and quantification calibrators (gBlocks listed in Tables 2 and 3, or control strains at 10^4^ and 10^3^ CFU/mL). McNemar’s test was used to compare serotype detection in the qPCR reaction-set with that of the Quellung method. Samples needed to be positive for both pneumococcal reference genes Lyt*A* and Pia*B* to be assigned a positive serotype. Where these pneumococcal genes were not detected, but a serotype-specific assay-set was positive, no serotype was assigned. Serotype designation by the ‘Fluidigm’ qPCR and Quellung were considered different where *p-*values were ≤ 0.05. Cohen’s kappa coefficient was used to determine the serotype-specific concordance of ‘Fluidigm’ qPCR and Quellung per clinical sample. Kappa values of < 0.20, 0.21–0.40, 0.41–0.60, 0.61–0.80 and 0.81–1.00 were considered as poor, fair, moderate, good, and excellent agreement, respectively. Analyses were done in Stata version 13.0, including applying relevant algorithms for serotype assignment.

### Repeatability and reproducibility

The repeatability (intra-assay variance) was determined using the standard deviation (SD) for the variance in Cq of gBlocks run in triplicate within the same IFC at 10^3^ gene equivalents. The reproducibility (inter-assay variance) was defined as the standard deviation for Cq variance for the gBlocks between four different IFCs.

### Ethical approval

Ethical approval was obtained from the Medical Human Research Ethics Committee (HREC) of the University of Witwatersrand for the initial sample collection (Soweto Cohort HREC: M090115; Agincourt Cohort HREC: M090114). Written informed consent was obtained from all legal guardians of the participants as part of the original study in which samples were obtained. Molecular methods and the use of samples in this analysis was approved by the HREC of the University of Witwatersrand (HREC: M170314). All methods were performed in accordance with the relevant local and international guidelines and regulations for Good Clinical Practice.

## Results

### Comprehensive serotyping reaction-set

**Figure 2:**
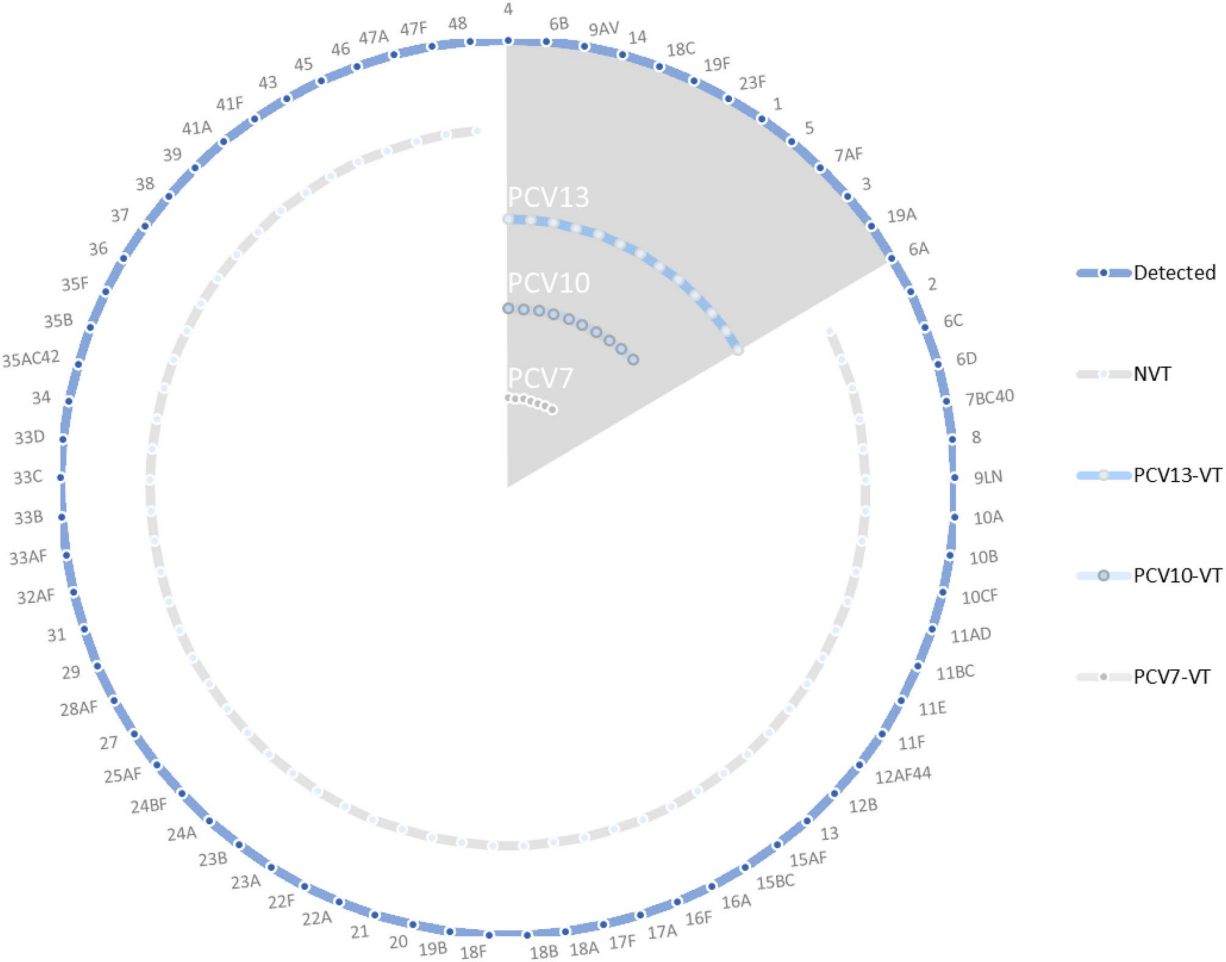
Summary web of the 92 pneumococcal serotypes (57 individual serotypes, and 35 serotypes within 16 serogroups) detected by the ‘Fluidigm’ reaction-set. Highlighted in grey are PCV7 serotypes: 4, 6B, 9A/V, 14, 18C, 19F and 23F; PCV10 serotypes: PCV7 serotypes and 1, 5, 7A/F; PCV13 serotypes: PCV10 serotypes and 3,6A, & 19A. NVT: serotypes not included in PCV13.

A total of 96 assay-sets were included in the final ‘Fluidigm’ qPCR reaction-set. The qPCR could detect 92 pneumococcal serotypes and differentiate these into 35 serotypes in 16 groups and 57 individual types and identify 15 other bacterial species (Fig. 2, Supplementary Table S4).

### Analytical performance of the ‘Fluidigm’ qPCR

Amplification efficiency across the reaction-set was excellent for 91 assay-sets that were within the prescribed range of 90–110% (Table 1). For these assay-sets, the data points were accurately predicted by the linear equation of the regression curves (r^2^ > 0.98, Table 1), allowing for relative quantification of bacterial load using the slope of the linear equation. The linear dynamic range for these assay-sets was at least 5-log fold. Further, Levy-Jennings plots constructed using assay-set specific reference calibrators were within ± 2.0 SD of the mean Cq value across all plates. This validated the use of the designed calibrators (gBlocks). The assay-sets for the detection of *E. coli*, *Pneumocystis jiroveci*, *S. algalactiae*, the *Xisco* gene of *S. pneumoniae* and the Bacterial 16S ribosomal RNA gene (listed in Supplementary Table S1) did not perform well within this reaction-set, as the efficiency was < 90% or > 110% or r^2^ < 0.98 (Supplementary Table S5).

**Table 1:** Analytical performance of 93 optimized assay-sets in the high-throughput BioMark HD nano-fluidic real-time PCR (‘Fluidigm’).

Assay-set	Calibrator	Efficiency (%) [− 1 + 10^(−1/m)^]	LOD (g-Block)	Linear equation	r^2^
1	St 1	98	10^1^	y = − 3.3761x + 24.236	1.00
2	St 2	95	10	y = − 3.4361x + 26.784	1.00
3	St 3	108	10^1^	y = − 3.1449x + 22.45	1.00
4	St 4	100	10^1^	y = − 3.32x + 28.393	1.00
5	St 5	99	10^1^	y = − 3.3421x + 25.415	1.00
6A/B/C/D(F/G/H)	St 6B	100	10^1^	y = − 3.327x + 30.357	0.99
6A/C	St 6C	97	10^2^	y = − 3.4039x + 30.277	0.97
6C/D	St 6C	97	10	y = − 3.384x + 27.769	0.99
7A/F	St 7F	95	10^1^	y = − 3.4605x + 26.292	1.00
7B/C/40	St 7C	102	10^1^	y = − 3.2729x + 27.595	0.99
8	St 8	110	10^2^	y = − 3.1071x + 24.264	1.00
9A/L/N/V	St 9A	102	10^1^	y = − 3.268x + 25.114	1.00
9A/V	St 9 V	101	10^1^	y = − 3.3014x + 24.495	0.99
9L/N	St 9N	99	10	y = − 3.3583x + 28.397	1.00
10A	St 10A	99	10	y = − 3.3555x + 24.534	1.00
10A/B	St 10B	100	10^1^	y = − 3.3263x + 23.409	0.99
10C/F	St 10C	96	10^2^	y = − 3.4327x + 28.19	1.00
11A/B/C/D/E/F	St 11A	102	10^1^	y = − 3.2846x + 26.706	0.99
11A/D	St 11D	91	10^2^	y = − 3.5656x + 29.722	1.00
11B/C	St 11B	100	10^2^	y = − 3.3137x + 25.745	1.00
11F	St 11F	103	10^2^	y = − 3.2412x + 30.921	0.99
12A/B/F/44/46	St 12B	100	10^2^	y = − 3.3217x + 22.458	1.00
12B	g-Block	97	10^1^	y = − 3.4084x + 26.027	1.00
13	St 13	105	10	y = − 3.2135x + 24.699	1.00
14	St 14	100	10^2^	y = − 3.3232x + 26.189	0.99
15A/B/C/F	St 15A	97	10	y = − 3.3908x + 26.255	1.00
15A/F	St 15A	109	10^2^	y = − 3.119x + 23.396	0.99
15B/C	St 15B	98	10^2^	y = − 3.3707x + 25.31	0.99
16A	g-Block	102	10	y = − 3.2674x + 24.3	0.99
16F	St 16F	104	10^2^	y = − 3.2321x + 25.116	1.00
16F/18F/28AF	St 18F	108	10^1^	y = − 3.1525x + 24.213	0.99
17A	St 17A	91	10^1^	y = − 3.5508x + 23.693	1.00
17A/F	St 17F	99	10^1^	y = − 3.3389x + 26.05	0.98
18A/B/C/F	St 18C	99	10^2^	y = − 3.3484x + 22.148	1.00
18B/C/F	St 18C	98	10	y = − 3.363x + 23.995	1.00
18C/F	St 18C	98	10^2^	y = − 3.3743x + 31.732	0.99
19A	St 19A	94	10^2^	y = − 3.483x + 23.801	1.00
19B/F	St 19B	108	10^2^	y = − 3.1345x + 20.812	1.00
19F	g-Block	110	10^1^	y = − 3.1075x + 26.863	1.00
20	St 20	99	10^2^	y = − 3.3405x + 25.595	0.99
21	St 21	103	10^2^	y = − 3.2409x + 27.273	0.99
22A/F	St 22F	98	10^2^	y = − 3.3701x + 26.527	1.00
22F	St 22F	95	10^2^	y = − 3.438x + 30.669	0.98
23A/B/F	St 23B	94	10^2^	y = − 3.4648x + 29.288	0.98
23B	St 23B	97	10^2^	y = − 3.3888x + 28.063	1.00
23F	St 23F	102	10^1^	y = − 3.2754x + 22.908	0.99
24A	St 24A	94	10^1^	y = − 3.4846x + 25.594	0.99
24B/F	St 24F	103	10^2^	y = − 3.2571x + 25.548	1.00
25A/F	St 25F	90	10^1^	y = − 3.5977x + 23.92	0.99
25A/F/38	St 25A	93	10^2^	y = − 3.5069x + 24.259	1.00
27	St 27	95	10^2^	y = − 3.4509x + 24.242	0.99
28AF	g-Block	92	10^2^	y = − 3.517x + 24.565	1.00
29	g-Block	105	10^2^	y = − 3.2145x + 26.526	1.00
31	g-Block	104	10	y = − 3.2831x + 22.833	1.00
32A/F	St 32A	98	10	y = − 3.3804x + 22.641	0.99
33A/F/37	St 33A	100	10	y = − 3.3257x + 23.074	0.99
33B/C	St 33C	97	10	y = − 3.406x + 25.401	1.00
33C	St 33C	96	10^1^	y = − 3.4155x + 25.436	1.00
33D	g-Block	109	10	y = − 3.1097x + 25.098	1.00
34/17A	St 34	97	10	y = − 3.4042x + 24.341	1.00
34/37/17A	St 37	100	10	y = − 3.3282x + 27.087	1.00
35A/C/42	St 35C	100	10	y = − 3.3161x + 25.966	0.99
35B	St 35B	103	10^1^	y = − 3.251x + 27.268	0.98
35F/47F	St 35F	90	10^2^	y = − 3.5757x + 24.976	0.98
36	St 36	102	10^1^	y = − 3.2814x + 26.331	1.00
39	g-Block	106	10	y = − 3.462x + 21.292	1.00
41A	St 41A	101	10	y = − 3.3039x + 24.147	1.00
41F	g-Block	94	10^2^	y = − 3.4843x + 22.181	0.98
43	g-Block	105	10	y = − 3.1997x + 27.245	0.99
45	St 45	90	10	y = − 3.6009x + 26.052	1.00
46	g-Block	98	10^1^	y = − 3.3655x + 21.273	1.00
47AF	St 47F	95	10^1^	y = − 3.442x + 24.074	0.99
48	St 48	91	10	y = − 3.5707x + 24.586	0.99
*Acinetobacter baumanii*	*A. baumanii*	94	10^1^	y = − 3.4785x + 26.193	0.99
*Bordetella holmesii* (h*IS*)	g-Block	91	10^2^	y = − 3.5491x + 29.271	1.00
*B. pertussis/holmesii* (*IS481*)	*B. pertussis*	90	10^2^	y = − 3.5933x + 25.235	0.99
*B. parapertussis* (p*IS*)	*B. parapertussis*	95	10	y = − 3.4358x + 21.956	0.99
*B. pertussis/bronchiseptica/parapertussis* (Ptx*S*)	*B. parapertussis*	97	10	y = − 3.3903x + 22.329	0.99
*Haemophilus influenzae* (Bex*A*)	*H. influenzae*, type b	93	10^1^	y = − 3.513x + 25.169	1.00
*H. influenzae* (Bex*B*)	*H. influenzae*, type b	102	10^1^	y = − 3.2784x + 26.106	1.00
*H. influenzae, type b*	*H. influenzae*, type b	97	10^2^	y = − 3.3953x + 22.967	0.99
*H. influenzae* (Ig*A1*)	*H. influenzae*, type b	97	10	y = − 3.3905x + 21.628	1.00
*Klebsiella pneumoniae*	g-Block	99	10	y = − 3.3499x + 26.09	0.98
*Morexella catarrhalis*	*M. catarrhalis*	97	10^1^	y = − 3.4079x + 26.561	0.99
*Neiserria lactamica*	*N. lactamica*	102	10^1^	y = − 3.2662x + 25.872	0.98
*Neiserria meningitidis*	*N. meningitidis*	99	10^2^	y = − 3.3496x + 24.255	0.98
*Staphylooccus aureus*	*S. aureus*	91	10^1^	y = − 3.5547x + 29.696	1.00
*Streptococcus oralis*	g-Block	108	10^1^	y = − 3.1503x + 26.386	1.00
*Streptococcus pneumoniae* (Lyt*A*)	St 4	99	10^1^	y = − 3.3582x + 26.927	1.00
*S. pneumoniae* (Pia*B*)	St 4	97	10^1^	y = − 3.394x + 26.863	0.99
*S. pneumoniae* (Ply)	St 4	96	10^1^	y = − 3.4107x + 26.927	1.00
*S. pyogenes*	g-Block	99	10	y = − 3.3488x + 25.05	1.00

### Analytical sensitivity, specificity, and quantification of density

The analytical sensitivity was high for 91 included assay-sets (LOD: 10–100 gene equivalents, Table 1). The assay-sets performed consistently, with Cq values of replicates (n = 3) amplified concurrently within a reaction-plate (intra-assay) or in consecutive reaction-plates (inter-assay) within ± 0.1 SD of the mean. Of the 96 assay-sets within the ‘Fluidigm’ qPCR, 94 assay-sets were specific to their targeted bacterial control calibrators, and there was no observed cross-reactivity between assay-sets. Two reaction-sets, namely 10A and 33B detected other serotypes within the same group—i.e., 10B and 33C respectively. These assay-sets were thus renamed accordingly—10A was renamed 10A/B and 33B was renamed 33B/C. A serotyping algorithm based on the detection pattern of all assay-sets against all targeted bacterial strains was applied to dissect some individual serotypes, namely: 6A, 6B, 6C, 6D, 10A, 11E, 12A/F/44, 18A, 18B, 18C, 18F, 19B, 22A, 23A, 38, 37, 33AF, 35F, 47A, 47F, non-typeable *S. pneumoniae*, non-type-b *H. influenzae*, non-typeable *H. influenzae*, *B. pertussis*, *B. holmesii* and *B. parapertussis* (Table 2). Assay-sets for serotypes 10B and 33C had been included within the reaction-set and therefore the specificity of serotype allocation by the algorithm was not affected by cross reacting serotypes, as 10A and 33B were not detected by these respective assay-sets. For example, serotype 10A would be detected by assay-set 10A and not assay-set 10B and would therefore be assigned serotype 10A; whereas serotype 10B would be detected by assay-set 10A and assay-set 10B and therefore be assigned serotype 10B.

**Table 2:** The algorithm for designating bacterial strains based on analytical specificity of assay-sets to their targets.

	6A	6B	6C	6D	10A	10B	11E	12A/F/44	18A	18B	18C	18F	19B	19F	22A	22F	23A
6A/B/C/D	**+**	**+**	**+**	**+**	−	−	−	−	−	−	−	−	−	−	−	−	−
6A/C	**+**	−	**+**	−	−	−	−	−	−	−	−	−	−	−	−	−	−
6C/D	−	−	**+**	**+**	−	−	−	−	−	−	−	−	−	−	−	−	−
10A/B	−	−	−	−	**+**	−	−	−	−	−	−	−	−	−	−	−	−
10B	−	−	−	−	**+**	**+**	−	−	−	−	−	−	−	−	−	−	−
11A/B/C/D/E/F	−	−	−	−	−	−	**+**	−	−	−	−	−	−	−	−	−	−
11A/D	−	−	−	−	−	−	−	−	−	−	−	−	−	−	−	−	−
11B/C	−	−	−	−	−	−	−	−	−	−	−	−	−	−	−	−	−
11F	−	−	−	−	−	−	−	−	−	−	−	−	−	−	−	−	−
12A/B/F/44/46	−	−	−	−	−	−	−	**+**	−	−	−	−	−	−	−	−	−
12B	−	−	−	−	−	−	−	−	−	−	−	−	−	−	−	−	−
16F	−	−	−	−	−	−	−	−	−	−	−	−	−	−	−	−	−
16F/18F/28AF	−	−	−	−	−	−	−	−	−	−	−	**+**	−	−	−	−	−
17A	−	−	−	−	−	−	−	−	−	−	−	−	−	−	−	−	−
17A/34/37	−	−	−	−	−	−	−	−	−	−	−	−	−	−	−	−	−
18A/B/C	−	−	−	−	−	−	−	−	**+**	**+**	**+**	−	−	−	−	−	−
18BCF	−	−	−	−	−	−	−	−	−	**+**	**+**	**+**	−	−	−	−	−
18CF	−	−	−	−	−	−	−	−	−	−	**+**	**+**	−	−	−	−	−
19B/F	−	−	−	−	−	−	−	−	−	−	−	−	**+**	**+**	−	−	−
19F	−	−	−	−	−	−	−	−	−	−	−	−	−	**+**	−	−	−
22AF	−	−	−	−	−	−	−	−	−	−	−	−	−	−	**+**	**+**	−
22F	−	−	−	−	−	−	−	−	−	−	−	−	−	−	−	**+**	−
23A/B/F	−	−	−	−	−	−	−	−	−	−	−	−	−	−	−	−	**+**
23B	−	−	−	−	−	−	−	−	−	−	−	−	−	−	−	−	−
23F	−	−	−	−	−	−	−	−	−	−	−	−	−	−	−	−	−
25A/F	−	−	−	−	−	−	−	−	−	−	−	−	−	−	−	−	−
25A/F/38	−	−	−	−	−	−	−	−	−	−	−	−	−	−	−	−	−
28A/F	−	−	−	−	−	−	−	−	−	−	−	−	−	−	−	−	−
33A/F/37	−	−	−	−	−	−	−	−	−	−	−	−	−	−	−	−	−
33B/C	−	−	−	−	−	−	−	−	−	−	−	−	−	−	−	−	−
33B	−	−	−	−	−	−	−	−	−	−	−	−	−	−	−	−	−
34	−	−	−	−	−	−	−	−	−	−	−	−	−	−	−	−	−
35F/47F	−	−	−	−	−	−	−	−	−	−	−	−	−	−	−	−	−
46	−	−	−	−	−	−	−	−	−	−	−	−	−	−	−	−	−
47A/F	−	−	−	−	−	−	−	−	−	−	−	−	−	−	−	−	−
IS481	−	−	−	−	−	−	−	−	−	−	−	−	−	−	−	−	−
PtxS1	−	−	−	−	−	−	−	−	−	−	−	−	−	−	−	−	−
hIS1001	−	−	−	−	−	−	−	−	−	−	−	−	−	−	−	−	−
pIS1001	−	−	−	−	−	−	−	−	−	−	−	−	−	−	−	−	−
IgA1	−	−	−	−	−	−	−	−	−	−	−	−	−	−	−	−	−
BexA	−	−	−	−	−	−	−	−	−	−	−	−	−	−	−	−	−
BexB	−	−	−	−	−	−	−	−	−	−	−	−	−	−	−	−	−
Hin−b	−	−	−	−	−	−	−	−	−	−	−	−	−	−	−	−	−

	25A/F	33A/F	33B	33C	35F	37	38	47A	47F	*B.pertussis*	*B.holmesii*	*B.parapertussis*	*B.bronchiseptica*	*H. influenzae*	*H.influenzae−b*	NTHI	
6A/B/C/D	−	−	−	−	−	−	−	−	−	−	−	−	−	−	−	−	
6A/C	−	−	−	−	−	−	−	−	−	−	−	−	−	−	−	−	
6C/D	−	−	−	−	−	−	−	−	−	−	−	−	−	−	−	−	
10A/B	−	−	−	−	−	−	−	−	−	−	−	−	−	−	−	−	
10B	−	−	−	−	−	−	−	−	−	−	−	−	−	−	−	−	
11A/B/C/D/E/F	−	−	−	−	−	−	−	−	−	−	−	−	−	−	−	−	
11A/D	−	−	−	−	−	−	−	−	−	−	−	−	−	−	−	−	
11B/C	−	−	−	−	−	−	−	−	−	−	−	−	−	−	−	−	
11F	−	−	−	−	−	−	−	−	−	−	−	−	−	−	−	−	
12A/B/F/44/46	−	−	−	−	−	−	−	−	−	−	−	−	−	−	−	−	
12B	−	−	−	−	−	−	−	−	−	−	−	−	−	−	−	−	
16F	−	−	−	−	−	−	−	−	−	−	−	−	−	−	−	−	
16F/18F/28AF	−	−	−	−	−	−	−	−	−	−	−	−	−	−	−	−	
17A	−	−	−	−	−	−	−	−	−	−	−	−	−	−	−	−	
17A/34/37	−	−	−	−	−	+	−	−	−	−	−	−	−	−	−	−	
18A/B/C	−	−	−	−	−	−	−	−	−	−	−	−	−	−	−	−	
18BCF	−	−	−	−	−	−	−	−	−	−	−	−	−	−	−	−	
18CF	−	−	−	−	−	−	−	−	−	−	−	−	−	−	−	−	
19B/F	−	−	−	−	−	−	−	−	−	−	−	−	−	−	−	−	
19F	−	−	−	−	−	−	−	−	−	−	−	−	−	−	−	−	
22AF	−	−	−	−	−	−	−	−	−	−	−	−	−	−	−	−	
22F	−	−	−	−	−	−	−	−	−	−	−	−	−	−	−	−	
23A/B/F	−	−	−	−	−	−	−	−	−	−	−	−	−	−	−	−	
23B	−	−	−	−	−	−	−	−	−	−	−	−	−	−	−	−	
23F	−	−	−	−	−	−	−	−	−	−	−	−	−	−	−	−	
25A/F	**+**	−	−	−	−	−	−	−	−	−	−	−	−	−	−	−	
25A/F/38	**+**		−	−	−	−	+	−	−	−	−	−	−	−	−	−	
28A/F	−	−	−	−	−	−	−	−	−	−	−	−	−	−	−	−	
33A/F/37	−	**+**	−	−	−	+	−	−	−	−	−	−	−	−	−	−	
33B/C	−	−	**+**	**+**	−	−	−	−	−	−	−	−	−	−	−	−	
33B	−	−	**+**	−	−	−	−	−	−	−	−	−	−	−	−	−	
34	−	−	−	−	−	−	−	−	−	−	−	−	−	−	−	−	
35F/47F	−	−	−	−	**+**	−	−	−	**+**	−	−	−	−	−	−	−	
46	−	−	−	−	−	−	−	−	−	−	−	−	−	−	−	−	
47A/F	−	−	−	−	−	−	−	**+**	**+**	−	−	−	−	−	−	−	
IS481	−	−	−	−	−	−	−	−	−	+	+	−	−	−	−	−	
PtxS1	−	−	−	−	−	−	−	−	−	+	−	−	−	−	−	−	
hIS1001	−	−	−	−	−	−	−	−	−	−	+	−	−	−	−	−	
pIS1001	−	−	−	−	−	−	−	−	−	−	−	+	+	−	−	−	
IgA1	−	−	−	−	−	−	−	−	−	−	−	−	−	+	+	+	
BexA	−	−	−	−	−	−	−	−	−	−	−	−	−	+	+	−	
BexB	−	−	−	−	−	−	−	−	−	−	−	−	−	+	+	−	
Hin−b	−	−	−	−	−	−	−	−	−	−	−	−	−	−	+	−	

### Diagnostic sensitivity, specificity, and agreement of the ‘Fluidigm’ qPCR

**Figure 3:**
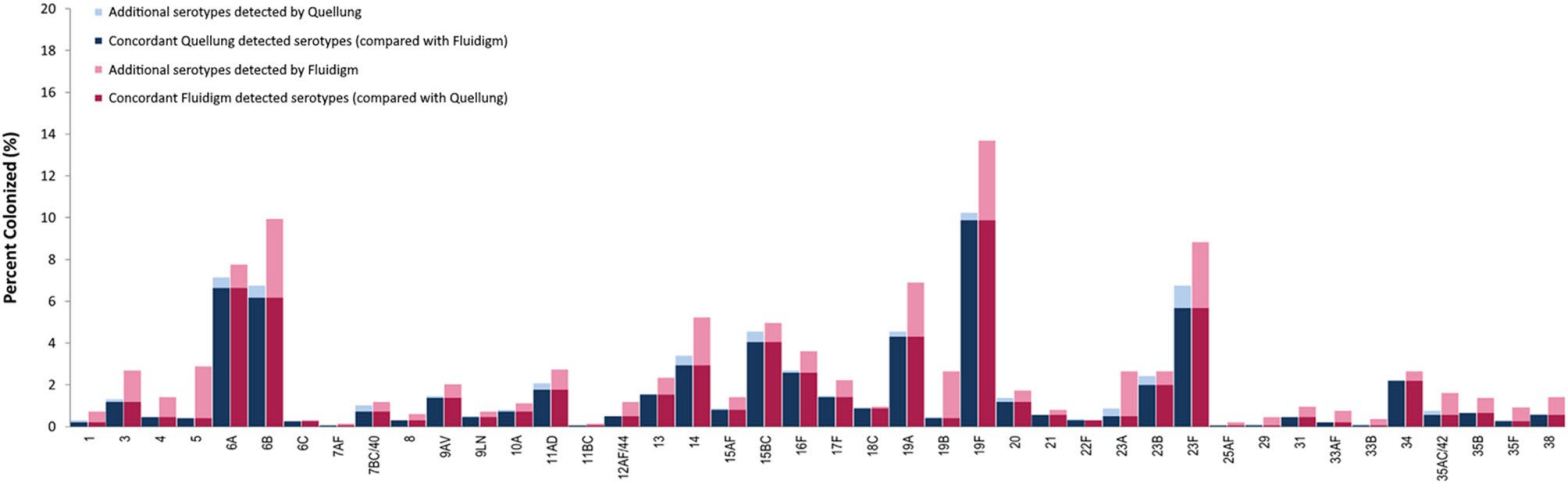
Prevalence of individual *Streptococcus pneumoniae* serotypes detected by the ‘Fluidigm’ qPCR reaction-set compared with the culture based Quellung method. Serotypes detected in samples by both ‘Fluidigm’ and Quellung are classified as concordant, whereas samples detected by only one method are classified as additional serotypes.

As the diagnostic performance was compared with the Quellung method, this was only applied to pneumococcal serotypes. Further, the diagnostic performance of the ‘Fluidigm’ could not be assessed for assay-sets that targeted bacterial species or pneumococcal serotypes that were not previously detected in archived clinical samples using culture-based methods (listed in footnote of Table 3). The ‘Fluidigm’ qPCR method was capable of correctly classifying pneumococcal serotypes compared with the referent standard (Quellung) and the receiver operator curve area under the curve (ROC-AUC) was greater than 0.73 for all compared assay-sets (Table 3). ‘Fluidigm’ qPCR distinguished targets with > 80% sensitivity (Table 3) in the 1 973 archived clinical samples for 36 assay-sets. The diagnostic sensitivity for six assay-sets (1; 7B/C/40; 23A; 29; 33B and 35A/C/42) was lower (50–74%, Table 3) as the prevalence of the targeted serotypes detected by Quellung was ≤ 1% whereas additional targeted serotypes were detected using ‘Fluidigm’ qPCR (Fig. 3). The remaining assay-sets could not be assessed as explained above. The diagnostic specificity was high (> 95%) for all assay-sets where targeted serotypes were previously detected by Quellung (Table 3).

**Table 3:** Diagnostic performance of the ‘Fluidigm’ high-throughput BioMark HD nano-fluidic real-time qPCR Fluidigm.

Serogroup/type	Sensitivity (%)	Specificity (%)	Correctly classified (%)	ROC AUC*	Cohen’s Kappa	Concordant samples	Discordant Fluidigm	Discordant Quellung	p-value^†^
1	66.7	99.4	99.4	0.83	Fair (0.40)	4	10	2	0.039
3	88.5	98.4	98.3	0.93	Moderate (0.57)	23	30	3	< 0.001
4	100	99.0	99.0	1.00	Moderate (0.48)	9	19	0	< 0.001
5	100	97.5	97.5	0.99	Fair (0.24)	8	49	0	< 0.001
6A	92.9	98.8	98.4	0.96	Excellent (0.88)	131	22	10	0.050
6B	91.7	95.9	95.7	0.94	Good (0.72)	122	74	11	< 0.001
6C	100	99.9	100	1.00	Excellent (0.91)	5	1	0	1.00
7A/F	100	99.9	99.9	1.00	Moderate (0.50)	1	2	0	0.500
7B/C/40	70.0	99.5	99.2	0.85	Good (0.65)	14	9	6	0.607
8	100	99.6	99.7	1.00	Good (0.67)	6	6	0	0.031
9A/V	93.1	99.3	99.2	0.96	Good (0.78)	27	13	2	0.007
9L/N	90.0	99.7	99.7	0.95	Good (0.75)	9	5	1	0.219
10A	87.5	99.5	99.5	0.94	Good (0.73)	14	8	2	0.109
11A/D	85.4	99.0	98.7	0.92	Good (0.73)	35	19	6	0.015
11B/C	100	99.9	99.9	1.00	Moderate (0.50)	1	2	0	0.500
12A/F/44	100	99.3	99.3	1.00	Moderate (0.60)	10	13	0	< 0.001
13	96.8	99.1	99.1	0.98	Good (0.78)	30	16	1	< 0.001
14	86.6	97.6	97.3	0.92	Good (0.67)	58	45	9	< 0.001
15A/F	94.1	99.3	99.3	0.97	Good (0.71)	16	12	1	0.003
15B/C	88.9	99.0	98.6	0.94	Excellent (0.84)	80	18	10	0.185
16F	96.2	98.9	98.9	0.98	Excellent (0.82)	51	20	2	< 0.001
17F	96.6	99.1	99.1	0.98	Good (0.76)	28	16	1	< 0.001
18C	94.4	99.9	99.9	0.97	Excellent (0.92)	17	2	1	1.00
19A	94.4	97.2	97.2	0.96	Good (0.74)	85	51	5	< 0.001
19B	88.9	97.7	97.7	0.93	Fair (0.26)	8	44	1	< 0.001
19F	96.5	95.7	95.8	0.96	Good (0.80)	195	75	7	< 0.001
20	85.2	99.4	99.2	0.92	Good (0.75)	23	11	4	0.119
21	100	99.7	99.8	1.00	Excellent (0.81)	11	5	0	0.063
22F	85.7	100	100	0.93	Excellent (0.92)	6	0	1	1.00
23A	58.8	97.8	97.5	0.78	Fair (0.28)	10	42	7	< 0.001
23B	81.3	99.3	98.9	0.90	Good (0.77)	39	13	9	0.524
23F	84.2	96.6	95.8	0.90	Good (0.71)	112	62	21	< 0.001
25A/F	100	99.8	99.9	1.00	Fair (0.40)	1	3	0	0.250
29	50.0	99.5	99.5	0.75	Poor (0.18)	1	8	1	0.039
31	100	99.4	99.5	1.00	Good (0.64)	9	10	0	0.002
33A/F	100	99.4	99.4	1.00	Moderate (0.42)	4	11	0	0.001
33B	50.0	99.7	99.7	0.75	Fair (0.22)	1	6	1	0.125
34	97.7	99.5	99.5	0.99	Excellent (0.89)	43	9	1	0.022
35A/C/42	73.3	98.9	98.7	0.86	Moderate (0.46)	11	21	4	< 0.001
35B	100	99.2	99.3	1.00	Good (0.65)	13	14	0	< 0.001
35F	83.3	99.3	99.3	0.91	Moderate (0.41)	5	13	1	0.002
38	91.7	99.1	99.1	0.95	Moderate (0.55)	11	17	1	< 0.001

There were no samples for which serotypes 2; 10B; 10C/F; 17A; 18A; 18B; 18F; 22A; 24A/B/F; 28A/F; 33D; 36; 37; 41; 43; 45; 47A/F and 48 were detected with the referent standard Quellung method for comparison of diagnostic performance.

*ROC AUC: Receiver operator curve, area under curve, ^†^McNemar’s Test.

There was good concordance between culture and ‘Fluidigm’ qPCR, with the majority of the assay-sets detecting the same serotype by both methods (kappa 0.61–0.8) with the exception of serotype/groups 1, 5 and 23A that had a fair concordance (kappa 0.41–0.60) and serotypes 7A/F, 11B/C, 12A/F/44, 33A/F, 35A/C/42, 35F, and 38 that had a moderate concordance (kappa 0.21–0.40). Where concordance was rated as fair or moderate, this was due to the detection of these serotypes in additional samples by ‘Fluidigm’ qPCR compared with Quellung (serotypes 5; 23A; 12A/F/44; 35A/C/42 and 38), and further, where there were less than five total samples detected by the Quellung method (serotypes 1; 7A/F; 11B/C; 33A/F and 35F) (Fig. 3). For one assay-set (serotype 29) the concordance was rated as poor (κ < 0.20). For this target, ‘Fluidigm’ qPCR detected one out of two samples serotyped as 29 using the culture based Quellung method, however, a further eight samples that were negative using Quellung, were serotyped as 29 using ‘Fluidigm’ qPCR. ‘Fluidigm’ qPCR was more sensitive in detecting all respective serotypes/groups compared to culture (McNemar’s test: *p* < 0.05; Table 3) except for serotypes 6C, 7A/F, 7B/C/40, 9L/N, 10A, 11B/C, 15B/C, 18C, 20, 21, 22F, 23B, 25A/F and 33B, where there was no difference in classification detected between the two methods (McNemar’s test: *p* > 0.05; Table 3). The ‘Fluidigm’ reaction-set was able to identify an additional 39.1% (826/2113) serotypes above those detected by culture for the compared assay-sets in Table 3, and conversely culture detected 132 (9.3% of 1419) serotypes not identified by ‘Fluidigm’ qPCR (Table 3, Fig. 3).

### Quantification of serotypes and bacterial density

**Figure 4:**
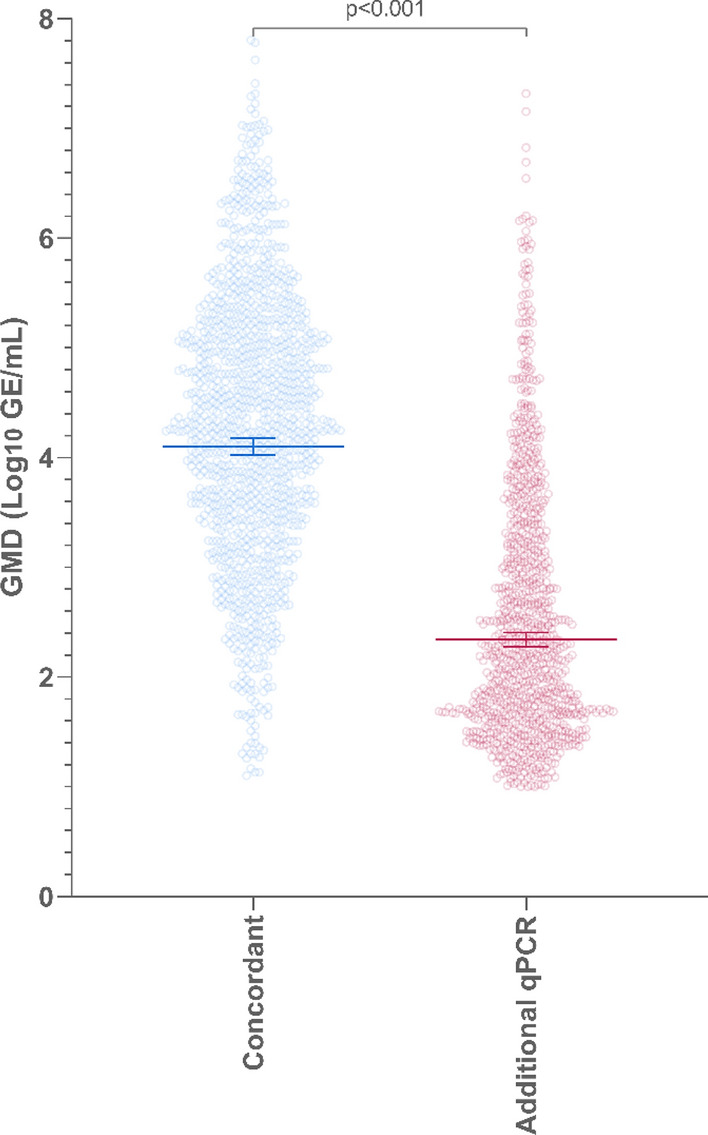
The Geometric Mean Density (GMD) expressed as Log10 Gene Equivalents (GE)/mL determined by ‘Fluidigm’ qPCR. Concordant serotypes (blue) were detected by both the referent standard culture based Quellung method and ‘Fluidigm’. Additional serotypes are those that were detected by ‘Fluidigm’ qPCR only (red).

The GMD (Geometric Mean Density) for all serotypes and bacterial targets are presented in Supplementary Table S6, and summarised in Supplementary Fig. S2, and the hierarchy of co-colonising pneumococcal serotypes is presented in Supplementary Table S7. Out of the additional serotypes detected by ‘Fluidigm’ qPCR, 72.6% (831/1 144) were co-colonising, or lower density serotypes relative to the primary, or highest density serotype. The GMD of the additional serotypes detected by ‘Fluidigm’ qPCR was lower than where the serotype designation was concordant between ‘Fluidigm’ qPCR and Quellung [2.3 (95% Confidence Interval/CI: 2.2–2.4) and 4.1 (95% CI: 4.0–4.2); Fig. 4].

There was excellent agreement between the pneumococcal density calculated from the Lyt*A* and Pia*B* assay-sets (line of bias: − 0.2) when compared using Bland–Altman plots (Supplementary Fig. S3A). Further, only 6.9% (58/836) of the data points were outside of the narrow 95% CI (− 1.7 to 1.3). The agreement between average pneumococcal density (Lyt*A* and Pia*B*) and the sum of all serotype density (all serotype-specific assay-sets) was acceptable (line of bias: − 1.2, Supplementary Fig. S3B) with 5.8% (45/776) of the data outside of the 95% CI (− 6.7 to 4.0). In 7.8% (65/836) of samples positive for pneumococcus, no serotypes were detected, and in a further 3.6% (30/836) the difference between the pneumococcal density and the total serotype density was above the upper CI of the Bland–Altman plot.

## Discussion

We have developed and validated a nanofluidic qPCR reaction-set that is sensitive, specific, and reproducible for the accurate detection and quantification of 92 pneumococcal serotypes and other colonizing bacteria within 96 specimens (including calibrators) per reaction-plate. The performance of the assay-sets described here is comparable with other high-throughput reaction-reaction sets to detect pneumococcal carriage, including in the Standard BioTools ‘Fluidigm’ platform^32,33^ and in TaqMan Array Cards^15^ for LOD (< 10^3^ CFU/ml); efficiency (90–110%); linear dynamic range (fivefold) and linearity (R^2^ > 0.98). Notably, the detection of additional serotypes (39.1%) by the ‘Fluidigm’ qPCR panel described here with 96 assay sets, is comparable to the ‘Fluidigm’ panel described previously (also 39.1%), that included 48 assay sets^32^. Compared with the gold-standard culture based-Quellung method, our reaction-set was 98.8% accurate for the classification of pneumococcal serotypes. Further, the average sensitivity across the qPCR reaction-set was 89.1% compared with Quellung.

Previous molecular serotyping techniques have detected common serotypes; however, other emerging or uncommon NVT were not detected. Expanding the array of serotypes detectable by higher throughput methods has become increasingly important as NVT replace VT in carriage^41–43^. It is also important to detect and assign bacterial load or density to multiple serotype carriage (co-colonization) directly from clinical specimens without an intervening culture step, which is an advantage of molecular techniques over standard culture-based methods^44^. Previously, incomplete serotyping has been described as a disadvantage of molecular serotyping in our setting (South Africa) where serotype prevalence differs from other geographic regions^45,46^. To the best of our knowledge, the ‘Fluidigm’ qPCR method described here is the most comprehensive high-throughput quantitative molecular serotyping reaction-set developed to date.

Notably, Pai et al.^25,26^ developed a conventional multiplex PCR method to determine 29 common serogroups/types, sequentially narrowed down to 40 singular serotypes. Azzari et al.^21^ used real-time PCR to detect 21 serotypes. Sakai et al.^29^ then designed 27 new assay-sets to detect 72 serotypes/groups. Pholwat et al.^15^ optimized a PCR-based Microfluidic TaqMan array card (TAC) covering 74 serotypes in up to seven clinical samples per card. Sakai et al.^30^ further developed a qPCR reaction-set to detect 46 individual serotypes and 33 serotypes within 20 serogroups. In the ‘Fluidigm’, Dhoubhadel et al.^33^ used SYBR Green chemistry to detect 50 serotypes (16 individual serotypes and 13 sub-groups). Messoudi et al.^31^ adapted Pai et al.^26^ assay-sets into a sequential real-time multiplex PCR to detect 40 serotypes or groups using Locked Nucleic Acid (LNA) modified probes. Together, these studies have furthered development into molecular serotyping and provided useful assay-sets for use in our ‘Fluidigm’ qPCR that we have further optimized within multiplex STA pools to detect and quantify 92 pneumococcal serotypes, distinguishing to 16 serogroups and 57 individual serotypes. Prior to our study, modified probes have not been utilized in a high throughput system such as micro- or nano-fluidic PCR systems or recommended in the Standard BioTools ‘Fluidigm’ platform. Our study has shown that these modified probe strategies work well in high-throughput ‘Fluidigm’ nano-fluidic qPCR.

Due to the aetiological link between high bacterial load, transmission risk and the invasive potential of serotypes, quantification of bacterial load in carriage studies is important to assess vaccine impact. Apart from serotypes 12F, 8, 24F, 33F, 38 and 10A, NVT involved in carriage serotype replacement have been shown to have low invasive potential according to case-to-carrier ratios analyses^47,48^; however, molecular studies have shown that high bacterial density may predict invasive potential of these colonizing serotypes. Out of the additional serotypes contained in PCV15 (22F, 33F) and PCV20 (8, 10A, 11A, 12F and 15BC), ‘Fluidigm’ qPCR identified an additional 0.3% of serotype 8; 0.4% of 10A; 1.0% of 11A/D; 0.7% of 12A/F/44; 0.9% of 15B/C and 0.6% of 33A/F compared with culture. A molecular study (multi-site: South Africa, Brazil, Mali, Cambodia and France) comparing the nasopharyngeal bacterial load of invasive isolates with asymptomatic carriage isolates for synonymous serotypes, found a fivefold higher mean bacterial load in invasive isolates (263.4 × 10^5^ CFU/ml; ± 57.07 × 10^5^ vs. 49.9 × 10^5^ CFU/ml ± 67.4 × 10^5^)^31^.

Bacterial prevalence may also be intrinsically linked to bacterial load that increases the risk of onward transmission. For example, serotype specific bacterial load and prevalence were compared in a Vietnamese study that included children under 5 years-of-age admitted to hospital with acute respiratory infection (ARI) and healthy age-matched children. Here, high bacterial load per serotype was associated with higher prevalence in both ARI cases (Spearman's ρ = 0.44, n = 186; *p* < 0.0001) and healthy children (Spearman's ρ = 0.41, n = 115; *p* < 0.0001).

Our reaction-set did not include the assay-sets previously published for 19F-Atypical, 19C and 23A; however, we did not find any indications in the in silico analysis that these assay-sets do not detect their respective targets, and these assay-sets have worked well in other studies^15,29,30^. Serotype 23A was still detected in our reaction-set using an algorithm that combines the results of the serogroup 23 (23A/B/F) assay-set and the serotype 23B- and 23F-targeted assay-sets whereby a sample positive for 23A/B/F but negative for 23B and 23F would be ascribed 23A.

While *LytA* and *PiaB* in combination are described as sensitive and specific for pneumococcal detection, and the density calculated using these assay sets is concordant, Tavares et al.^49^ have since recommend using the putative transcriptional regulator gene SP2020 combined with *LytA* for increased specificity. These four assay-sets (19F-Atypical, 19C, 23A and SP2020) should be further validated and included in the reaction-set to increase the detection to 94 serotypes, 16 serogroups and 59 individual serotypes. The assay-sets for the detection of *E. coli*, *P. jiroveci*, *S. agalactiae*, the *Xisco* gene of *S. pneumoniae* and the Bacterial 16S ribosomal RNA gene were not well optimized within this reaction-set and may therefore only be used for qualitative detection of targets.

Challenges exist for distinguishing within some serogroups to singular serotypes by our reaction-set. These include that a high degree of cross-reactivity between genetically similar serotypes limits discrimination at serotype level. For example, serotypes 7A/F and 9A/V differ genetically by only a single nucleotide in a difficult to target region (low GC content) with runs of homogonous base pairs that would result in mis-priming for all high-throughput strategies described here^2^. These will require more specialist strategies that do not fit within our uniform thermal profile. Further, some serotypes interconvert in situ and may not be clinically characterized easily into separate serotypes, such as serotypes 11A and 11E, 15B and 15C, or 35B and 35D, which exist on a ‘spectrum’ due to incomplete loss or gain of acetylation or transferases^50,51^. The latter two serogroups, although they contain molecularly distinct serotypes, have problematic clinical distinction, and therefore should be interpreted with caution.

Other limitations to this study include that our method requires experienced personal and specialized equipment (Standard BioTools, ‘Fluidigm’) that is not affordable in many settings; however, this method can be easily adapted to other real-time qPCR platforms. Where serotypes are not distinguished to individual serotypes, the bacterial load for serotypes detected within groups could be over-estimated if there is co-carriage of more than one serotype within a serogroup, for example serogroup 12A, 12F and serotype 44, or serotypes 7B, 7C and serotype 40. As colonization by most of these serotypes is not high in our setting, this is not expected to have a very relevant impact. The discrimination of these serotypes individually will be important in settings where these serotypes are in circulation or to assess emergence of non-vaccine types in the post-vaccine era.

The referent standard Quellung method is limited to the detection of higher density and dominant colonizers, whereas our ‘Fluidigm’ qPCR can detect multiple concurrent colonization, including at lower density. This is due to the inclusion of a comprehensive serotyping method (assay-sets covering most serotypes) and a high analytical sensitivity to detect low density colonizers. These benefits of qPCR translate to diagnostic comparisons of these methods under-estimating the diagnostic sensitivity and the concordance of ‘Fluidigm’ qPCR compared with the culture based Quellung methods, especially where the prevalence of serotypes detected by the referent standard is low. This was indeed the case for serotypes 1; 7B/C/40; 23A; 29; 33B and 35A/C/42.

A further limitation for diagnostic comparison in our study includes the use of retrospective samples that have undergone multiple freeze–thaw cycles and have been stored for around 10 years, which can affect the ability of any diagnostic method to accurately detect pathogens. The Quellung method was undertaken in 2010 (soon after sample collection) and detected an additional 9.3% of serotypes that were not detected by ‘Fluidigm’ qPCR. In contrast, the analytical sensitivity and specificity of the ‘Fluidigm’ qPCR was demonstrated across the reaction-set when newly prepared culture or gBlock calibrators were used. Additionally, the ‘Fluidigm’ qPCR reaction-set could detect 39.1% more serotypes that were missed by Quellung. Here, the density (GMD) of the additional serotypes detected by ‘Fluidigm’ qPCR was around two times lower than for serotypes concordantly detected by ‘Fluidigm’ qPCR and Quellung and most (72.6%) were sub-dominant serotypes that would be less likely to be detected by the Quellung method. Nonetheless, our method was able to serotype the 1 973 selected samples with good concordance and sensitivity compared with Quellung.

The use of qPCR-based serotyping relies on detection of short target sequences within a larger capsular polysaccharide synthesis locus, to assign serotypes. The recent description of serotype 14-like pneumococci highlights a limitation of short genetic identifiers. In Papua New Guinea, four NPS isolates from hospitalized children with acute lower respiratory tract infection were assigned serotype 14 (an invasive VT) by qPCR. These isolates were ‘non-typeable’ (non-encapsulated) using the Quellung, latex agglutination and PneumoCaT methods. The authors describe a divergent serological profile and the ability to escape vaccine-elicited immunity to capsular serotype 14, despite partial genetic similarity. Genomic sequencing of the 14-like isolates revealed mutations that interrupt the capsular biosynthetic loci, whereas the short PCR targeted serotype 14 sequence was intact^52^. Additionally, small changes within target sequences, such as single nucleotide polymorphisms (SNPs) are not easily discerned using standard qPCR methods, whereas these single point genetic changes can alter the capsular structure, as exemplified by serotypes 6A and 6B^53^. The implication from these two examples is that in some cases, molecular serotyping may not identify antigenic diversity or new serotypes, and alternate serotyping methods should be used to resolve discrepancies.

In 7.8% of samples where both Lyt*A* and Pia*B* were detected, no serotype was detected. Within the context of our method, the differential detection of pneumococcus and serotypes may indicate the presence of either non-typeable (NT) pneumococci or, less likely, serotypes not included in our reaction-set. In a further 3.8% of pneumococcal positive samples the difference in the pneumococcal density and the total serotype density was above the upper bound confidence interval of the Bland–Altman plot, which may be a useful strategy to identify NT pneumococci present in co-colonization with other serotypes. This method of detecting NT pneumococci in co-colonisation using the confidence intervals of the Bland–Altman plots would however underestimate NT present at lower density.

A limitation of our method involves the detection of capsular serotype target regions that have been acquired by non-pneumococcal species present in the nasopharynx. While we included that samples need to be positive for both Lyt*A* and Pia*B*, where non-pneumococcal serotype homologues are present in co-colonization with true pneumococcal serotypes, these would be assigned as pneumococcal serotypes. An example of this phenomenon is *Streptococcus mitis* that express a serotype 1 capsule^54^. In target assay-sets where qPCR concordance with Quellung was rated as poor, fair or moderate using Cohen’s kappa (serotypes 1; 5; 7A/F; 11B/C; 12A/F/44; 23A; 11B/C; 29; 33A/F; 35A/C/42; 35F and 38), this was due to the detection of these serotypes in additional samples by qPCR. When detected in co-colonisation, we cannot exclude that these additional serotypes detected are always true pneumococci. In the Bland–Altman plot for the agreement between average pneumococcal density and serotype density, where the total density of multiple concurrent colonising serotypes was higher than the total pneumococcal density and outside of the lower bound 95% CI, this may indicate the presence of non-pneumococcal serotype homologues. Here we propose additional sequencing analysis be undertaken where necessary for the confirmation of serotypes in co-colonization, where other non-pneumococcal *Streptococci* are detected.

One of the large benefits of our study is the description of performance per assay-set according to the minimum information for publication of quantitative real-time PCR experiments (MIQE) guidelines^55^ and the large sample size of Quellung-serotyped isolates available for validation of our method.

Although to set-up the reaction-set within the Standard BioTools ‘Fluidigm’ qPCR platform is initially expensive, advantages include a large reduction of labor costs and waiting time due to the high number of data points, as many samples are tested against many different serotypes run within one plate. Our method is relatively fast to perform and can yield results of 92 serotypes and 15 bacterial targets for 180 samples (two IFC runs) within one day. The cost per nasopharyngeal sample is around USD $36 which is highly favorable, considering the comprehensive serotyping and quantitative information generated per sample. The concatenated reaction-set within the Standard BioTools ‘Fluidigm’ qPCR will be a benefit in large carriage studies. The method should be further validated for other clinical specimens, such as blood, sputum, cerebrospinal fluid, and other sites where pneumococcus may be found.

## Supplementary Information

Supplementary Information.

## Data Availability

The datasets generated and/or analysed during the current study and the Stata codes are available from the corresponding authors on reasonable request.
