# Supplementary material for: Optimization of a high-throughput nanofluidic real-time PCR to detect and quantify of 15 bacterial species and 92 Streptococcus pneumoniae serotypes
Source: Sci Rep. 2023 Mar 21;13:4588. doi: 10.1038/s41598-023-31820-4 (PMC10030628; doi:10.1038/s41598-023-31820-4)
Supplement: Supplementary file 1 — Supplementary Information. [file 41598_2023_31820_MOESM1_ESM.docx]

Supplementary Material

**Optimization of a high-throughput nanofluidic real-time PCR to detect and quantify 15 bacterial species and 92 Streptococcus pneumoniae serotypes**

Sarah L. Downs ^1,2*^, Shabir. A. Madhi ^1,2^, Lara Van der Merwe ^1,2^, Marta. C. Nunes ^1,2^ and Courtney P. Olwagen ^1,2*^

^1^ South African Medical Research Council, Vaccines and Infectious Diseases Analytics Research Unit, School of Pathology, Faculty of Health Sciences, University of the Witwatersrand, Johannesburg, South Africa.

^2^ Department of Science and Technology/National Research Foundation, South African Research Chair Initiative in Vaccine Preventable Diseases, Faculty of Health Sciences, University of the Witwatersrand, Johannesburg, South Africa.

*** Correspondence:**Corresponding Authors
[sarah.downs@wits-vida.org](mailto:sarah.downs@wits-vida.org) ; [courtney.olwagen@wits-vida.org](mailto:courtney.olwagen@wits-vida.org)

Keywords: Pneumococcus (*Streptococcus pneumoniae*)_1_, PCV (Pneumococcal Conjugate Vaccine)_2_, Molecular Diagnostics_3_, Infectious Disease_4_, quantitative PCR_5_, carriage_6_.

**Supplementary Table 1**: Oligonucleotide sequences for the ‘Fluidigm’ qPCR reaction-set (96 assay-sets) for detection of 92 pneumococcal serotypes, 15 other bacterial and one fungal species including the use of Dual Priming Oligonucleotides (DPO; n=2), Locked Nucleic Acid probes (LNA; n=3) and internally quenched probes (n=8)

| **Assay-set name** | **Forward primer (5' to 3')** | **Reverse primer (5' to 3')** | **Probe (5' to 3')** | **Gene Target** | **Reference** |
| --- | --- | --- | --- | --- | --- |
| 1 † | CGTGCGGTAATTGAAGCTATGA | TGTGGCCCCAGCAACTCT | FAM-TGCTTGCCCTTGTATAGGGT-NFQ | wch*D* | Azzari *et al.* 2010 [1] |
| 2 ‡ | TTATGGACTGGCTGATGGTTCTC | AAATCCTGACCCAATAATAGCCTTT | FAM-AGGTCAACG/ZEN/TATTGGAAC  TCTTAGAAATTGGGAAA-IABkFQ | wzy | Pholwat *et al.* 2016 [2] |
| 3 † | GGTCAGCAGAAAGTATGCATTGG | TCGTTTATCCAGGGTCTGATGA | VIC-TATTGGATGTGGT  TTATCGTGAAGA-NFQ | tnp | Azzari *et al.* 2010 [1] |
| 4 † | TGGGATGACATTTCTACGCACTA | CCGTCGCTGATGCTTTATCA | FAM-TCCTATTGGATG  GTTAGTTGGTGA-NFQ | wzy | Azzari *et al.* 2010 [1] |
| 5 † | TTACGGGAGTATCTTATGTCTTTAATGG | CAGCATTCCAGTAGCCTAAAACTAGA | VIC-TTGTCTCAGCAACT  CTATTTGGCTGTGGG-NFQ | wzy | Azzari *et al.* 2010 [1] |
| 6A/B/C/D  (F/G/H) † | AAGTTTGCACTAGAGTATGGGAAGGT | ACATTATGTCCRTGTCTTCGATACAAG | VIC-GTTCTGCCCTGAGCAACTGG-NFQ | wci*P* | Azzari *et al.* 2010 [1] |
| 6A/C ‡ | CATTGCTAGAGATGGTTCCTTC  AGTTGATATTGATAAAGATTCG  GGAGACATGTCCAAACTGGC | CGATACAAGACCAGTTGC | FAM-GTTTGCACT/ZEN/AGAGTA  TGGGAAGGTGTTGT-IABkFQ | wci*P*α | Downs *et al*. 2021 [3] |
| 6C/D † | TTGGGATGATTGGTCGTATTAG | CTCTTCAATTAGTTCTTCAGTTCG | FAM-CCACGCAATTCGCCATC-NFQ | wci*N*_β_ | Azzari *et al.* 2010 [1] |
| 7A/F † | GATGGCATGTGGCAAACCA | TTTGCCCTCCTTAATCATTTCAC | FAM-TTGGCTATCGGCATGGTGGT-NFQ | wcw*H* | Azzari *et al.* 2010 [1] |
| 7B/C/40 ‡ | TCCAGATATAGTCATTCCCAATCAG | AAAGAAGGTAAATCCCATGATGAATT | FAM-TCCCTCATTATCGATTA  CTGACCCACCA-BHQ1 | wcx*U* | Pholwat *et al.* 2016 [2] |
| 8 † | CCACTCATCAGTTTCCCATATGTTT | TCAATAATTGAAGAAGCGAACGTT | FAM-TGATGGCAGAT  GGGTTGGGACGAG-NFQ | wzx | Azzari *et al.* 2010 [1] |
| 9A/L/N/V ‡ | TGGAATGGGCAAAGGGTAGTA | TCGGTTCCCCAAGATTTTCTC | FAM-TTAATCATGCTAACGG  CTCATCGA-BHQ1 | mna*A* | Olwagen *et al.* 2017 [4] |
| 9A/V‡ | AGGTATCCTATATACTGCTTTAGG | CGAATCTGCCAATATCTGAAAG | FAM-ACACATTGA  CAACCGCTACA-BHQ1 | wzx | Pholwat *et al.* 2016 [2] |
| 9L/N ‡ | CGTGGAATTTTCTATACTGCAATAGG | CTACTGCTACGATACCATATTCTACAG | FAM-CAATTCTTAG  CCGGATTCTCTC-BHQ1 | wzx | Pholwat *et al.* 2016 [2] |
| 10A/B † | CCTCTCCTATCAACTAT  TACTCATTATACTACCT | AATAACCATAAGTCC  CTAGATCATTCAAAG | VIC-TCATTACAACTCCCTA  TGTGACACGGGTCTTTT-NFQ | wcr*D* | Azzari *et al.* 2010 [1] |
| 10B † | AAATATGAGATTGGT  AAGGAATATTCTGG | GTCTTTTCACTTAAACGAATTCCATTC | FAM-AACGGATTCCAATGC  ACTCGGTAACT-NFQ | wcr*D* | Pholwat *et al.* 2016 [2] |
| 10C/F † | CGAGTTATGGATGTTCTTATTGGC | CCCAACCCCACTCTGTATTG | FAM-ACAGGGCAAGACTGT  GAATATTGTTCCA-NFQ | wcj*G* | Sakai *et al.* 2017 [5] |
| 11A/B/C/ D/F/(E) † | ACCGCATTTCTTATCGCACTATATT | TCTCCTTACCATCAAACATGTTAATCA | FAM-TGAATCAGTCTGACCGTTT-NFQ | wzx | Olwagen *et al.* 2017 [4] |
| 11A/D † | CGGCCCAGCTACATTTATGG | TGATCATTCACATGCTCACCAA | FAM-AAATACCAATAGTTGT  TCCGAGATTAAAGAAGT-NFQ | wch*K* | Pholwat *et al.* 2016 [2] |
| 11B/C † | TCAAATTTGGCGTATTGCTTATCA | TGATTATGAGCATAGTTGATCCCC | FAM-TCCGTGGCAAGATTCT  GGTGCTAAG-NFQ | wzy | Sakai *et al.* 2017 [5] |
| 11F † | TGGTCCAGCTACTTTTATGGC | TGATCATTCACATGCTCCCC | VIC-ACTCCAATAGTTGTTC  CGAGGCAAAAGA-NFQ | wch*K* | Pholwat *et al.* 2016 [2] |
| 12A/B/F/44/  46 † | GATTATTCGCTTGCCTCTTCATG | ATAGCCGAAATAAGCTTTCCAGAA | FAM-ATTTGTAAGCG  GACGTGCGATT-NFQ | mna*B* | Azzari *et al.* 2010 [1] |
| 12B † | GGTTGCTGATCAAAAGGTCTATG | AGGTTCAAAGTAAGATTTTTAGCAA | FAM-AGATAAAAATCTTTCC  AAATCATCAAAGTGA-NFQ | wzx | Pholwat *et al.* 2016 [2] |
| 13 † | TCGGATTTAGTAGTAACCCCATTGA | TTCTTGATTGAGGATGCATTTCC | VIC-AGTAGTAAGAG  ATCATATTCAAG-NFQ | wzy | Olwagen *et al.* 2017 [4] |
| 14 † | CGACTGAAATGTCACTAGGAGAAGAT | AATACAGTCCATCAA  TTACTGCAATACTC | VIC-TGTCATTCGTTTGCCA  ATACTTGATGGTCTC-NFQ | wch*L* | Azzari *et al.* 2010 [1] |
| 15A/B/C/F ‡ | TTGAATCAGGTAGATTGATTTCTGCTA | CTCTAGGAATCAAAT  ACTGAGTCCTAATGA | FAM-CTCCGGCTTT  TGTCTTCTCTGT-BHQ1 | wzx | Azzari *et al.* 2010 [1] |
| 15A/F ‡ | CGTTATTTAGTGAATTGCTATACTC | TCCCTGCAGAATAAGAATCTAC | FAM-TACtGCtGCtGCcAACA-BHQ1 | wzy | Messaoudi *et al.* 2016 [6] |
| 15B/C ‡ | TTTGCTACAGGTTTTAGTATTGAG | AAAGCAATATAAGAGGTATAGTTGG | FAM-CGCtACaATcATcCGCT-BHQ1 | wzy | Messaoudi *et al.* 2016 [6] |
| 16A † | GCTAGCAGGAACTTTTCTAGGG | TCCCTGTCCAAATCCGAAAC | FAM-CCCACGGGATGAATC  CATTATGGCG-NFQ | wcx*R* | Sakai *et al.* 2017 [5] |
| 16F † | GCAACTGGTATTTTTGATATTGGAGAA | CAAAGGAATGCCATGCCATA | FAM-AAAATGCTAAC  TTCGTTGGAGG-NFQ | wzy | Olwagen *et al.* 2017 [4] |
| 17A † | TGATTATGTCATTCGATTGCTTGG | AAATCCTAAAATTCCTGTTTGAAAAGC | FAM-ATTATGGGCGTG  GGTTACCGTAGG-NFQ | wzy | Sakai *et al.* 2017 [5] |
| 17F † | GTAAAGATTTCATGT  CCTATAAGGGAGAA | AGGCGTCCCTGTTTATGAGAAG | FAM-TTGTACATGGTCTGGATTT-NFQ | Wzx | Olwagen *et al.* 2017 [4] |
| 18A/B/C/F ‡ | CCTGTTGTTATTCACGCCTTACG | TTGCACTTCTCGAATAGCCTTACTC | FAM-AACCGTTGGC  CCTTGTGGTGGA-BHQ1 | wzy | Azzari *et al.* 2010 [1] |
| 18B/C/F ‡ | CAGGATTTCTAACTCTGATTGAA | AGCAAAATCTAACGTCCAGAG | FAM-CTTGTATGCTTATGG  TCTTTTCGATTA-BHQ1 | wci*X*_BCF_ | Downs *et al*. 2021 [3] |
| 18C/F ‡ | CCAAATTGGAGTGTTTTACAA  AGTATTAGCTCGATTTGCTGT  ACACGTCGACGCTTCAATTTCAGG | TCTTTCAAATACAACT  CTTAGATTTCCTTGTG | FAM-TGagtTTATTGATAATttcC-BHQ1 | wci*X*_CF_ | Downs *et al*. 2021 [3] |
| 18F:  16F/18F/28AF ‡ | TGGTTTCGGACTCTTTCGTGG | CTAAGATAGAAACTCCTTGTCCAATG | FAM-GGTTGTACGTGGAAT  CGGATTTGGTC-BHQ1 | wcx*M* | Downs *et al*. 2021 [3] |
| 19A † | TTCGACGACGTATCAGCTTCA | TCATTGAGAGCCTTAACCTCTTCA | VIC-ACCCAAAACGGTTG  ACGCATTATACT-NFQ | wzy | Azzari *et al.* 2010 [1] |
| 19B/F † | GGTCATGCGAGATACGACAGAA | TCCTCATCAGTCCCAACCAATT | VIC-ACCTGAAGGAGTAG  CTGCTGGAACGTTG-NFQ | wzy | Azzari *et al.* 2010 [1] |
| 19F † | CAGGTTCGGGAAATTGCAA | ATCTCTGCGCCATAAGCAATG | VIC-AGAAGTGGCAGATGATT-NFQ | wzh | This study |
| 20 † | AAAGATACTGGCTGAGGAGCTATCTATT | AGTCAAAAGTACTCAA  CCATTCTGATATATTC | VIC-AGGATAAGGTCTACT  TTGTGGGAGTTC-NFQ | wci*L* | Azzari *et al.* 2010 [1] |
| 21 † | CCATTTGAAGGACCAGTTGTTG | AAAAAGCCACTATCAGGAATACCAA | FAM-AATGGCATTGCTTCGTAAA-NFQ | wzy | Olwagen *et al.* 2017 [4] |
| 22A/F † | TCTATTAAATAACCC  ATTGGAATTGAAACG | TCGCAATTGAAGACCACATAAACTG | FAM-TCCGTAATTCGCTTA  TGGGCACATTCTCCA-NFQ | wcw*V* | Azzari *et al.* 2010 [1] |
| 22F ‡ | GAAGATTGTCCACCTTATATCC | TCGGCACAATCAAAATATC | FAM-CGGTTATTT/ZEN/  CACAAAAGACACGGTTGG-IABkFQ | wcw*A*_F_ | Downs *et al*. 2021 [3] |
| 23A/B/F † | GGTGGACTTTCCGATGCAA | CACTGTCAACAAAAATGAGGTAATCTC | FAM-AAATGTCGGTATAGATAAAG-NFQ | wchV | Olwagen *et al.* 2017 [4] |
| 23B † | TTGAAGAAATTGCTCCAGAAACAT | CCAAAAGACTAGCCTCAACCACTAA | FAM-TAGAGCTATTTATCTTT  CGTGGTTTT-NFQ | wzx | Pholwat *et al.* 2016 [2] |
| 23F † | TGCTATTTGCGATCCTGTTCAT | AGAGCCTCCGTTGTTTCGTAAA | FAM-TTTCTCCGGCA  TCAAACGTTAAG-NFQ | wzy | Azzari *et al.* 2010 [1] |
| 24A † | CTTGGAGTTGCTAATTATGGGAAG | ATCTCTTACACGTGCACACTC | FAM-CACAGCATATCGTAA  AATACCCGCA-NFQ | wzx | Pholwat *et al.* 2016 [2] |
| 24B/F ‡ | TCTGAAAGTAATTAG  TAAGATTAACGGAAG | TCCATCTACTTTTAAAATAGCTCCAAC | FAM-CCACAGTCCCAAAAT  TGTCAGCAACC-BHQ1 | wzy | Sakai *et al.* 2017 [5] |
| 25A/F † | ATACCAACTAGAATCAGCAGGAC | AAATGGAATATCTTT  TGATAATTTACTCGC | VIC-CCGCTGGACTTACTGCAATA-NFQ | wcy*E* | Pholwat *et al.* 2016 [2] |
| 25A/F/38 † | GTCTTACGTAGAACCTCTCTGGATGA | TGGTCCTACAAGCGACATGTG | FAM-TTGCCACAGATTTGG  AATATTTTGGTCGG-NFQ | wciI | Olwagen *et al.* 2017 [4] |
| 27 † | AGCGATTTAGCGACTGATATCC | TCTCAAAATCGATCTCGCGTG | FAM-TGTGGAAGGCGT  TTGAAGGTGACT-NFQ | wha*K* | Pholwat *et al.* 2016 [2] |
| 28A/F † | CAACTACAGGTATTTTTGATATCGGAG | GTTTACTACGTTTGTGAAGCGC | FAM-AGAAAATAGTAGGTT  GATTGGCGGTGCT-NFQ | wcx*P* | Sakai *et al.* 2017 [5] |
| 29 † | TTCGAGTTGTGCCGTTTTTACA | GGCGTACCCACCTCTAAAATTTT | VIC-TGAATCCTAGTCTTTTCTCTGCG-NFQ | wcr*J* | Pholwat *et al.* 2016 [2] |
| 31 † | GCAGAAGTTTTAAGTCACGGAC | AGCATTACAGATGTCACTAAGGG | FAM-CCCCCACGTAAAACCGCAAGG-NFQ | wzy | Pholwat *et al.* 2016 [2] |
| 32A/F † | GTACTTCCTGTTCTAGGCTTGG | CCCAGAGGAAAATAGCGTCTC | FAM-TTGTTCAAACC  CAACCACTGCTCC-NFQ | wzy | Sakai *et al.* 2017 [5] |
| 33A/F/37 † | GGAACTGGTTCAGCAACTATACG | GGTTCTAAGACCGTCTGAAATACC | FAM-TAGGACTTTTCTGCCATGCC-NFQ | wzy | Pholwat *et al.* 2016 [2] |
| 33B/C † | CCTGTTAGTGCACCTGTATTTAAC | GCATTCAAAACTCCTTCATCTCC | FAM-TTCGTTGTTCACGCCATTTA-NFQ | wci*N* | Pholwat *et al.* 2016 [2] |
| 33C † | CAGAGACAGTTTCAGCAAATCTTAG | AGCCTACACCTCTTATAAACGTTG | FAM-CCGTGTCCTATCCAC  AAACTTGTCTTCC-NFQ | Wzy | Sakai *et al.* 2017 [5] |
| 33D ‡ | CGTATAGTCTTGCGACATTTCA | TTCCACATGCGTTACCTCAC | FAM-CACAACTAG/ZEN/TTTTTTA  TCAAAAAGACCTTGGC-IABkFQ | wci*N* | Pholwat *et al.* 2016 [2] |
| 34/37/17A † | GGATACTATGTACGAACAGATGGACTTG | CTCACTAACTCGCCCGAATAAAC | FAM-CCGACTATACTCCATTTGA-NFQ | wci*B* | Olwagen *et al.* 2017 [4] |
| 34 † | CGGTGGAGTAGGTCAAGATG | GTCTGTTCTCCCCAATATACTGAG | FAM-ACGGAGCGCCAATG  TACTTGAATAGTT-NFQ | Wzy | Pholwat *et al.* 2016 [2] |
| 35A/C/42 † | TGTTTCAAGCTTCCCCTTTAGA | AAATGAAATCAAAGTATCACGTATCG | FAM-TTCAAAATACCCAG  GACACCCGTTCA-NFQ | wcr*K* | Pholwat *et al.* 2016 [2] |
| 35B † | GCATGGAGGTGGAGCATACA | TGTAAAGACTGCACAACTCGATATAAAA | FAM-CAATTTAAACAATATTAG  TAAAGCGCAGGTCAAGCAAA-NFQ | wcr*J* | Azzari *et al.* 2010 [1] |
| 35F/47F † | GTGGTCGTATATACT  TGATGAATAAATCG | ACATACAAATTATCA  ACATACAGATAGGTC | FAM-TTCAACTGGTCGTCCGAATA-NFQ | Wzy | Pholwat *et al.* 2016 [2] |
| 36 † | CTTGTCTATTCAGCCCTTCTGG | CGCGATTATATTGTAAATTGGGAACT | VIC-AGAATGCCCGCTACAATGAG-NFQ | Wzy | Pholwat *et al.* 2016 [2] |
| 39 † | CAAAAAAATGAACTA  ACTCAAATAGTAACG | ATACTGTAATTTTCTTGTTTATTTGCGG | FAM-AAGTCAGGCGTATTC  TTCACAAGGGAAA-NFQ | wcr*G* | Pholwat *et al.* 2016 [2] |
| 41A † | GCAAATAGATGTATCCCAGTTAACAC | GGTAGCTCTTTTGGTTTAATGTCC | FAM-CGACCGAATAGTCT  AGCTTCAAAGG-NFQ | wci*B* | Pholwat *et al.* 2016 [2] |
| 41F † | TTTTTGGGAGGAAGTGCTTTT | AACCGCTTTCTCATGATTCATAACT | FAM-CTTCTGTGCTA  ACAGTGGAGAT-NFQ | Wzx | Pholwat *et al.* 2016 [2] |
| 43 † | AGAGGCTACATCAAATAGTTGGC | GAATCACACCGTAACTTCCAAAG | FAM-TCCAATAGTACTCA  CCCCTACCGAGC-NFQ | Wzx | Pholwat *et al.* 2016 [2] |
| 45 † | TCTAGCTACTTGACTA  AAATATTTGAACTG | GACGAGTCGATTTCGCTGTAT | VIC-CTTTTAGTGACCTCGCTCCC-NFQ | Wzy | Pholwat *et al.* 2016 [2] |
| 46 † | CGAAGTTTTTATATCTCTATTGGTTTG | TATCCCAGGAACTGGACGAA | FAM-TCATTCTTTCTTCAAT  TCCTTTCTGA-NFQ | Wzy | Sakai *et al.*2015 [7] |
| 47A/F † | AGGAATTGGTAGAGAGTTTGTGG | GAAAGTTGAACCATCATCCGTC | FAM-CACTTGATGGA  ATGCCTGCTGCC-NFQ | whaI | Pholwat *et al.* 2016 [2] |
| 48 † | CAGGTTTTGCTTCATATGGGAG | ATCGGCCAAAAGTTATCATTAGC | FAM-CGCTGCTTATGTGTA  TTACTCTCCCCTG-NFQ | Wzy | Sakai *et al.* 2017 [5] |
| *Acintobacter baumanii* † | TTTAGCTCGTCGTATTGGACT | CCTCTTGCTGAGGAGTAATTTT | FAM-TGGCAATGCAG  ATATCGGTACCCA-NFQ | blaOXA  -51-like | Gadsby *et al.*, 2015 [8] |
| *Bordetella*  *holmesii* † | GGCGACAGCGAGACAGAATC | GCCGCCTTGGCTCACTT | FAM-CGTGCAGATAGGCTT  TTAGCTTGAGCGC-NFQ | hIS*1001* | Tatti *et al.*, 2011 [9] |
| *B. parapertussis* † | TCGAACGCGTGGAATGG | GGCCGTTGGCTTCAAATAGA | FAM-AGACCCAGGGCGCACGCTGTC-NFQ | pIS*1001* | Tatti *et al.*, 2011 [9] |
| *B. pertussis/*  *Holmesii* † | CAAGGCCGAACGCTTCAT | GAGTTCTGGTAGGTGTGAGCGTAA | FAM-CAGTCGGCCTT  GCGTGAGTGGG-NFQ | IS*481* | Tatti *et al.*, 2011 [9] |
| *B. pertussis/*  *bronchiseptica/ parapertussis* † | CGCCAGCTCGTACTTC | GATACGGCCGGCATT | FAM-AATACGTCGAC  ACTTATGGCGA-NFQ | ptx*S1* | Tatti *et al.*, 2011 [9] |
| *Escherichia coli* † | GTCCAAAGCGGCGATTTG | CAGGCCAGAAGTTCTTTTTCCA | FAM-ACGGCAGAGAAGGTA-NFQ | uid*A* | Lee *et al.,* 2006 [10] |
| *Haemophilus influenzae* † | CTCAGTTTCGTTTTATTACCA | CCAGTAACAACAAGGCTA | FAM-CGCATTCTTCTTCGTC  CATAACCTTC-NFQ | BexB | This Study |
| *H. influenzae* † | CTGAATTRGGYGATTATCTTTATGA | ACAATCAAAYTCAACHGAAAGHGA | FAM-AGGGATGAAA  GCYCGRCTTGCAT-NFQ | BexA | Maaroufi *et al,.* 2007 [11] |
| *H. influenzae* † | CAAAATTGCCAAGATTAAATGCTT | TGCTCGCCATACTGCACAA | FAM-CCTGCGGTTAAACC-NFQ | IgA1 | Olwagen *et al.* 2017 [4] |
| *H. influenzae,*  type b † | TGTTCGCCATAACTTCATCTTAGC | CTTACGCTTCTATCTCGGTGATTAATAA | FAM-CACAAAACTTCTCAT  TCTTCGAGCCTA-NFQ | Bcs3` | Maaroufi *et al.,* 2007 [11] |
| *Klebsiella pneumoniae* ‡ | AGGCCGAATATGACGAAT | GGTGATCTGCTCATGAA | FAM-ACTACCGT  CACCCGCCACA-BHQ1 | glt*A* | Gadsby *et al.*, 2015 [8] |
| *Morexella catarrhalis* † | CCGCTTTTACAACCACTGCTT | TGTATCGCCTGCCAAGACAA | FAM-CAGCTGTTAGCCAGCC-NFQ | tonB | Olwagen *et al.* 2017 [4] |
| *Neiserria*  *Lactamica* † | TTGCCCGAGAACCATTGTATC | GCGGTTCTTATCACGTTCTATATTTG | FAM-TATTGGAGCGGACTAAA-NFQ | lacZ | Olwagen *et al.* 2017 [4] |
| *Neiserria meningitidis* † | GCACACTTAGGTGATTTACCTGCAT | CCACCCGTGTGGATCATAATAGA | FAM-CATGATGGCACAGCA  ACAAATCCTGTTT-NFQ | SodC | Dolan Thomas *et al.,* 2011 [12] |
| *Pneumocystis jiroveci* ‡ | CCATCACATCTACGATTAC | GAACGAAATAACCATTGC | FAM-ACTCACATC  AACGAGGCGGT BHQ1 | Msg-A1 | Jensen, 2002 (Modified) [13] |
| *Staphylooccus aureus* † | GCTCAGCAAATGCATCACAAA | CACTATATACTGTTGGATCTTCAGAACCA | FAM-AGATAACGGCGTAAATA-NFQ | Tfp | Olwagen *et al.* 2017 [4] |
| *Streptococcus pneumoniae* ‡ | TCTTACGCAATCTAGCAGATGAAGC | GTTGTTTGGTTGGTTATTCGTGC | FAM-TTTGCCGAAAACG  CTTGATACAGGG- BHQ1 | LytA | McAvin *et al.*, 2001 [14] |
| *S. pneumoniae* ‡ | AGCAGGTGACTGGTAGGTAAC | CTCCTAATGCTGCTCC | FAM-CAGTTGCTT/ZEN/GCG  GTGCACTTG-IABkFQ | Xisco | This Study |
| *S. pneumoniae* † | AGCGATAGCTTTCTCCAAGTGG | CTTAGCCAACAAATCGTTTACCG | FAM-ACCCCAGCAAT  TCAAGTGTTCGCG-NFQ | Ply | Greiner *et al.*2001 [15] |
| *S. pneumoniae* ‡ | CATTGGTGGCTTAGTAAGTGCAA | TACTAACACAAGTTC  CTGATAAGGCAAGT | FAM-TGTAAGCGG/ZEN/AAAAG  CAGGCCTTACCC-IABkFQ | PiaB | Trzciński *et al.*2013 [16] |
| *S. pyogenes* † | GCACTCGCTACTATTTCTTACCTCAA | GTCACAATGTCTTGGAAACCAGTAAT | FAM-CCGCAACTCATCAAG  GATTTCTGTTACCA-NFQ | Spy | CDC 2008; Kodani, 2011 [17] |
| *S. algalactidae* † | GAACTCTAGTGGCTGGTGCATTG | GGAGTTGTCACTTGATCAGCATGT | FAM-ATTTTCACCAGCTGTATTAG-NFQ | Cfb | CDC [18] |
| *S. oralis* ‡ | ACCAGCAGATACGAAAGAAGCAT | AGGTTCGGGCAAGCGATCTTTCT | FAM-AAGGCTGCT/ZEN/GTT  GCTGAAGAAGT-IABkFQ | gtf*R* | Alvarez, 2013 [19] |
| Bacterial 16S ribosomal RNA gene ‡ | TCCTACGGGAGGCAGCAGT | GGACTACCAGGGTATCTAATCCTGTT | FAM-CGTATTACC/ZEN/GCG  GCTGCTGGCAC-IABkFQ | 16S | Nadkarni *et al.*, 2002 [20] |

Note: † Ordered from Thermofisher; ‡ ordered from Integrated DNA Technologies; NFQ is Nonfluorescent quencher; IABkFQ is an Iowa Black Fluorescent Quencher; BHQ1 is Black Hole Quencher 1.

**Supplementary Table 2**: External synthetic calibrator (gBlock) properties.

| **gBlock**™ **name** | **Assay-Set Targets** † | **Length (bp)** | **Amount**  **(ng/µl)** ‡ | **Gene equivalents (Copy number)** |
| --- | --- | --- | --- | --- |
| A1: Pool A gBlock, no. 1 | 1, 4, 5, 6A/B/C/D, 33D, LytA, 19F (Atypical), 7A/F, 8, 9A/L/N/V | 1088 | 8,09 | 1,36 x 10^10^ |
| A2: Pool A gBlock, no. 2 | 10C/F, 11A/D, 14, 20, Hib, 16F, 22F, 15B/C, 18A/B/C, 19B/F | 1147 | 6,73 | 1,07 x 10^10^ |
| A3: Pool A gBlock, no. 3 | 11F, 24A, 28A/F, 17A, 35F/47F, 41F, 12A/B/F/44/46, 23A/B/F, Eco, Sag, hIS1001, Nme | 1225 | 8,53 | 1,27 x 10^10^ |
| B1: Pool B gBlock, no. 1 | 3, 6A/C, 7B/C/40, 9A/V, 10B, 11A/B/C/E/F, 12B, 13, 15A/B/C/F, 16A, 34/37/17A, 18B/C/F, 19C | 1474 | 3,82 | 4,73 x 10^9^ |
| B2: Pool B gBlock, no. 2 | 19F, 22A/F, 23F, 25A/F, 33C, 35B, 46, 47A/F, Xis | 1023 | 7,71 | 1,38 x 10^10^ |
| B3: Pool B gBlock, no. 3 | BexA, Nla, Mca, Spy, Sau, 16S, PiaB, PtxS1, plS1001, IS481, Sor | 1122 | 5,13 | 8,34 x 10^9^ |
| C1: Pool C gBlock, no. 1 | 15A/F, Ply, 11B/C, 16F/18F/28A/F, 17F, 19A, 21, 23B, 24B/F, 25A/F/38, 27, 29 | 1284 | 7,04 | 1,00 x 10^10^ |
| C2: Pool C gBlock no. 2 | 31, 32A/F, 33A/F/37, 33B, 34, 35A/C/42, 45, 48, 2, 10A, 9L/N | 1252 | 5,28 | 7,70 x 10^9^ |
| C3: Pool C gBlock no 3 | Aba, Kpn, 23A, BexB, IgA1, Pji, 36, 39, 41A, 43, 6C/D. | 1160 | 4,11 | 6,47 x 10^9^ |

† Listed in order of position on gBlock; ‡ Average value of 3 successive Q-bit measurements

**Supplementary Table 3**: External synthetic calibrator (gBlock) sequences

| **gBlock name** | **gBlock sequence** |
| --- | --- |
| A1 | TACCAGACGATGCTTATCAATGACCCCTATATGTGTGATATCGTGCGGTAATTGAAGCTATGAGTTGTGCTTGCCCTTGTATAGGGTCTCCAGAGTTGCTGGGGCCACATCACCGCAGGTATCCCGTCGCCGGTTGGGATGACATTTCTACGCACTATCTTTCCTATTGGATGGTTAGTTGGTGATAGTTGATAAAGCATCAGCGACGGGGGTTGCTCTGGGTGCCATGAACTATTACGGGAGTATCTTATGTCTTTAATGGTTTGTCTCAGCAACTCTATTTGGCTGTGGGAATTCTAGTTTTAGGCTACTGGAATGCTGGCAGCGACGACCACCACACACACATGCAAGTTTGCACTAGAGTATGGGAAGGTGTTGTTCTGCCCTGAGCAACTGGTGCTACTTGTATCGAAGACATGGACATAATGTTAACCTATGAACTCGATCAGAGAACGTATAGTCTTGCGACATTTCATCCACAACTAGTTTTTTATCAAAAAGACCTTGGCGAGGTGAGGTAACGCATGTGGAACATACCAAGCATCTCCATCTAAGTTCTTACGCAATCTAGCAGATGAAGCTATTTGCCGAAAACGCTTGATACAGGGTATGCACGAATAACCAACCAAACAACTATTAATATGGTATATTTATGTCGCGTCCTTAGTTCTGGTTATTCGGGAATCCAGTTATGAAGGTGAGCTAACAGTGCGACACTTCGATTCGGTTCCTCATCCGCCCTACCGACAGTGGTACTAGAGGCGATGGCATGTGGCAAACCATAGTTGGCTATCGGCATGGTGGTGTTGTGAAATGATTAAGGAGGGCAAAAACGGTCTCCTAGCCACTCCAGCTATTCCCACTCATCAGTTTCCCATATGTTTCATTCTGATGGCAGATGGGTTGGGACGAGTAACGTTCGCTTCTTCAATTATTGATTGCCCACGCAATGGGGATGCTGGAATGGGCAAAGGGTAGTAAGTTAATCATGCTAACGGCTCATCGACGAGAAAATCTTGGGGAACCGATGGAGAGCCCGAGTTAAGGTGGAGCTCAAGTAAATGGTCTACCGACACTACTCTCAGAAG |
| A2 | TTCCACGGCTCAAGTAAATGGTCTTGATTAGTAAGGCAGGATGCATAAACCAGGAGGTACCGAGTTATGGATGTTCTTATTGGCTTGTGGAACAATATTCACAGTCTTGCCCTGTTCACAATACAGAGTGGGGTTGGGAGGGTAGATGTTATCATACACATGGCGGCCCAGCTACATTTATGGCAGTTATTGCTAAAGGAAAAATACCAATAGTTGTTCCGAGATTAAAGAAGTATTGGTGAGCATGTGAATGATCATCAGATGGATTGATACAACCCATGGCGACTGAAATGTCACTAGGAGAAGATATGTCATTCGTTTGCCAATACTTGATGGTCTCAGAGTATTGCAGTAATTGATGGACTGTATTATACCATGTGCCATCCACAGTTCGTAAAAGATACTGGCTGAGGAGCTATCTATTCAGGATAAGGTCTACTTTGTGGGAGTTCAGTCGAATATATCAGAATGGTTGAGTACTTTTGACTCCGTCTGCTGATGATTGCGCATGGGTACATCTGTTCGCCATAACTTCATCTTAGCACCACAAAACTTCTCATTCTTCGAGCCTAATTATTAATCACCGAGATAGAAGCGTAAGGGGGTACTAGAGTAGATCGACAATGGACATTTTCCAAATGAAGATGTCCACCTTATATCCAACAGAACGGTAGATATTTTGATTGTGCCGAAAGGTTTAGGAATAGCTGCATTAGAGCAACTGGTATTTTTGATATTGGAGAACAAAATGCTAACTTCGTTGGAGGTGTATGGCATGGCATTCCTTTGGGATTCTTGTTACCGTTAATTAGTTTGCTACAGGTTTTAGTATTGAGAAAGCGGATGATTGTAGCGATCCAACTATACCTCTTATATTGCTTTATGAATACTATTACTGGAACGCCTGTTGTTATTCACGCCTTACGTAACCGTTGGCCCTTGTGGTGGATGGAGTAAGGCTATTCGAGAAGTGCAACTTGAAGATGAAGCCTGTGGTCATGCGAGATACGACAGAAAGACCTGAAGGAGTAGCTGCTGGAACGTTGAAATTGGTTGGGACTGATGAGGAGACTAAATCACTCACAAAGAAAATTTAACGCTTGATGTGATGACCCCATAGTGTTCGAGGTCATTCCGAGTTATCCAG |
| A3 | GATGACCTTCGTGGCGTGCTAGTATGCATAGTGTTCGAGATGACCTGCTTCATGCCATGTGGTCCAGCTACTTTTATGGCTACTCCAATAGTTGTTCCGAGGCAAAAGAATGGGGAGCATGTGAATGATCATCTTGGAGTTGCTAATTATGGGAAGTCTGCGGGTATTTTACGATATGCTGTGCAGAGTGTGCACGTGTAAGAGATGATGAGGCATGCATATTAGTCAATGCAACTACAGGTATTTTTGATATCGGAGTAGAAAATAGTAGGTTGATTGGCGGTGCTCAGCGCTTCACAAACGTAGTAAACTAGTGAGTACGGGGTCTAGGCATCTGATTATGTCATTCGATTGCTTGGAACCTACGGTAACCCACGCCCATAATGAGCTTTTCAAACAGGAATTTTAGGATTTGCATATTGTTGGATATAGGTGGTCGTATATACTTGATGAATAAATCGGATTATTCGGACGACCAGTTGAACAGACCTATCTGTATGTTGATAATTTGTATGTCTCGCTCTAGGAATAATGCTCCTAGTTTTTGGGAGGAAGTGCTTTTACTTCTGTGCTAACAGTGGAGATATAGTTATGAATCATGAGAAAGCGGTTGCATTGATACGAAGAGAAGCATCGAAGATTATTCGCTTGCCTCTTCATGGATTTGTAAGCGGACGTGCGATTGTTCTGGAAAGCTTATTTCGGCTATTAAGGGTCTCCAGAGGTATTGTAGGTGGACTTTCCGATGCAAGAAATGTCGGTATAGATAAAGCGGAGATTACCTCATTTTTGTTGACAGTGTCATGAACGGTTGGTTATGTCCAAAGCGGCGATTTGGACGGCAGAGAAGGTACTGGAAAAAGAACTTCTGGCCTGGCAGGAGACTGCATCAGCTATCCTGGAACTCTAGTGGCTGGTGCATTGTATTTTCACCAGCTGTATTAGGTACATGCTGATCAAGTGACAACTCCAGGGCCCTCGATGCTGGGAGCCGGTGGCGACAGCGAGACAGAATCCCGTGCAGATAGGCTTTTAGCTTGAGCGCGAAGTGAGCCAAGGCGGCCACATGGTTACCCATGGCGATGATGCACACTTAGGTGATTTACCTGCATTCATGATGGCACAGCAACAAATCCTGTTTTCTCTATTATGATCCACACGGGTGGCACGATTATGCTAGTCATCACCGAATACGGTGTGGATACGTTAGCGTATGCGGCCTGCA |
| B1 | TTAACTTTAAGCTTGATGTGATGACCTTTTGCCATAGTGTTCGAGGTCATTGGTCAGCAGAAAGTATGCATTGGTTATTGGATGTGGTTTATCGTGAAGACCATCATCAGACCCTGGATAAACGAGCTGCACCTTAATCTTATCCGGTGCATTGCTAGAGATGGTTCCTTCAGTTGATATTGATAAAGATTATTTATATATAGAAAAACTGGCAGTTTGCACTAGAGTATGGGAAGGTGTTGTTGAGCAACTGGTCTTGTATCGAAGACATGGACATAATGTAACAACTAGTCATCATCCAGATATAGTCATTCCCAATCAGGGGTTGGTGGGTCAGTAATCGATAATGAGGGAAAAATTCATCATGGGATTTACCTTCTTTTAAGAGCGTTGTACTTGCAGAGGTTGAAGGTATCCTATATACTGCTTTAGGTATGTAGCGGTTGTCAATGTGTTTCTTTCAGATATTGGCAGATTCGGGAATAGGACCTGCAATAAAATATGAGATTGGTAAGGAATATTCTGGTCAACGGATTCCAATGCACTCGGTAACTTATGAATGGAATTCGTTTAAGTGAAAAGACATGTTGACATTCAGTAGTATGGAGATACCGCATTTCTTATCGCACTATATTTTGAATCAGTCTGACCGTTTGATGATTAACATGTTTGATGGTAAGGAGAAATTAGCTATATATAGTCTAGCTGGTTGCTGATCAAAAGGTCTATGTTTCACTTTGATGATTTGGAAAGATTTTTATCTAATTGCTAAAAATCTTACTTTGAACCTAGGATATGCTGAACGATATGGATCGGATTTAGTAGTAACCCCATTGAGAGTAGTAAGAGATCATATTCAAGGTGGAAATGCATCCTCAATCAAGAATGCCATTATAGGGGAGAGATCTTGAATCAGGTAGATTGATTTCTGCTATGTCTCCGGCTTTTGTCTTCTCTGTATTAGGAAATATCATTAGGACTCAGTATTTGATTCCTAGAGGATGTTAGAACTATGTGGGATGCTAGCAGGAACTTTTCTAGGGGCTATACGCCATAATGGATTCATCCCGTGGGACGTTTCGGATTTGGACAGGGAGACAGATCGAGATGATTATCGTGGCTTCTGGATACTATGTACGAACAGATGGACTTGACCGACTATACTCCATTTGAGACGTTTATTCGGGCGAGTTAGTGAGTTGTTGTACGCCTTATGTACATGGCATTTGCAGGATTTCTAACTCTGATTGAATACTTGTATGCTTATGGTCTTTTCGATTAACAACTCTGGACGTTAGATTTTGCTGTCAGCGACGTATTTACTATGAGTAAATGGTTTTCAGATTACTTGATAGCTCTGAGTTCATTATGTGGGGGCAGGAACAAATCTTGACCACTCTCATAAGGAACGTAATGAGACATGGACTTACTATAAGACTTGACAGTAGAAGGAAAAGTCAAATCTGTCTCTTAG |
| B2 | ATCTTCCTACAGGAAGGACGAAGCCAATATACAACCTTGTACGTCTTAGTAATGGATATACAGGTTCGGGAAATTGCAACAAGAAGTGGCAGATGATTTATCATTGCTTATGGCGCAGAGATGACGTATCTGGGGTGGATTGTATAGTCTATTAAATAACCCATTGGAATTGAAACGGCTTGGAGAATGTGCCCATAAGCGAATTACGGAACAGTTTATGTGGTCTTCAATTGCGAGGAGATTTGATTGAGGCAGACATTGCTATTTGCGATCCTGTTCATTCTTTCTCCGGCATCAAACGTTAAGGTTTACGAAACAACGGAGGCTCTATGTGAGATGTTTTATCACCGCTTATTTATGATACCAACTAGAATCAGCAGGACACCCGCTGGACTTACTGCAATACTGCGAGTAAATTATCAAAAGATATTCCATTTTTGAGGTGCGCTATTCCCTGTCACAGTCAGAGACAGTTTCAGCAAATCTTAGATGGAAGACAAGTTTGTGGATAGGACACGGTCCAACGTTTATAAGAGGTGTAGGCTATGCTCAGGGAGGATTATTTCTATTTCTAATCAAGGCATGGAGGTGGAGCATACAAGACAATTTAAACAATATTAGTAAAGCGCAGGTCAAGCAAATATTTTATATCGAGTTGTGCAGTCTTTACAAAGGTGCCTGGTTTGGCAAGTCGAAGTTTTTATATCTCTATTGGTTTGTAATCATTCTTTCTTCAATTCCTTTCTGAGCTTCGTCCAGTTCCTGGGATATTAGTAGCGATGCTGTGAATTATAGTAAAGAGGAATTGGTAGAGAGTTTGTGGATCCACTTGATGGAATGCCTGCTGCCCCGACGGATGATGGTTCAACTTTCTCAGTTTAATAGTTTAGTAAAGCAGGTGACTGGTAGGTAACACAGCAGTTGCTTGCGGTGCACTTGTAGGAGCAGCATTAGGAGCCACTGGTCTCTTAGGAACTGTAACTTTGACAAATATGGAGCAAATGATATTTTACCTGGTTTAAC |
| B3 | ACTGTGAAGAAATACTCTTCGTATGATGTTGATCCAGACTATGTGACTCGACAGAATTTTCTGAATTAGGTGATTATCTTTATGAACAGGGATGAAAGCTCGGCTTGCATTTCTCTTTCTGTTGAATTTGATTGTCGCCAACAAACTATTGACAACTTCTGTTATTTTGCCCGAGAACCATTGTATCGTATTGGAGCGGACTAAAGCAAATATAGAACGTGATAAGAACCGCCAAGCCTCATGAGGCGCTGACGGCGGTGAGTGCCGCTTTTACAACCACTGCTTTTGCAGCTGTTAGCCAGCCTGTTGTCTTGGCAGGCGATACAGTGGTCAGTGATCGCCGCACTCGCTACTATTTCTTACCTCAAATCCGCAACTCATCAAGGATTTCTGTTACCAACTATTACTGGTTTCCAAGACATTGTGACCGGGCAATACGCAGCTACTAGTAGCTCAGCAAATGCATCACAAACAGATAATGGCGTAAATAGGTGGTTCTGAAGATCCAACAGTATATAGTGCTGAGAGGATGGGACACGGTCCCTCCTACGGGAGGCAGCAGTTGTGCCAGCAGCCGCGGTAATACGGCAAACAGTAGATACCCTGGTAGTCCCATCAAGCATAAGCCATATAAGTTTTGCAATCATTGGTGGCTTAGTAAGTGCAAGCTGTAAGCGGAAAAGCACTTGCCTTATCAGGAACTTGTGTTAGTATGTAAGGTACCTCATAACTGCCGCCAGCTCGTACTTCGACAATACGTCGACACTTATGGCGATAAATGCCGGCCGTATCGGATCTTTACCTTGCGATGCGTAACTGCCGTTTCGAACGCGTGGAATGGCGAAGACCCAGGGCGCACGCTGTCCGTTCTATTTGAAGCCAACGGCCTGCAATCATCCGATGTACGTGGCCAAGGCCGAACGCTTCATAGTTACGCTCACACCTACCAGAACTCAACAGTCGGCCTTGCGTGAGTGGGATGCTCAAGAGCCTGCTAAACCAGCAGATACGAAAGAAGCATCAAAGGCTGCTGTTGCTAGAAAGATCGCTTGGAACCTTTTAAGCACGCCGTAAACGACAATCAATACCATATTCCTTATTTAGAAGCCAGTATTGAGGCTC |
| C1 | GATATGATAGTTGATGGAGACAGGTGACATATGACTACACTGTATCAAGTTGTTCATATATTCGTTATTTAGTGAATTGCTATACTCCGATGTTGGCAGCAGCAGTAATGTAGATTCTTATTCTGCAGGGAGAGCTCACATTAGTTTAATGCTCAGCGATAGCTTTCTCCAAGTGGAAGACCCCAGCAATTCAAGTGTTCGCGGAGCGGTAAACGATTTGTTGGCTAAGTGGCATCAAGAAATGGCCGCTATCAAATTTGGCGTATTGCTTATCACTTCCGTGGCAAGATTCTGGTGCTAAGATGGGGATCAACTATGCTCATAATCAGCATGCCAGATGTACCTTTGTCGTATTGGTTTCGGACTCTTTCGTGGATGGTTGTACGTGGAATCGGATTTGGTCCGCATTGGACAAGGAGTTTCTATCTTAGCAAAGCGTAAATTATTTGTTGGCAACTAGTAAAGATTTCATGTCCTATAAGGGAGAAGTTTGTACATGGTCTGGATTTATGATTGCTTCTCATAAACAGGGACGCCTAGTTAGTATCATCACCAGATATCTTTCGACGACGTATCAGCTTCACCACCCAAAACGGTTGACGCATTATACTTATGAAGAGGTTAAGGCTCTCAATGACAATTAGCAATAGATAATGGAGTTGATATGCCATTTGAAGGACCAGTTGTTGAAATGGCATTGCTTCGTAAAGTTGGTATTCCTGATAGTGGCTTTTTTATTGTAATTGGTATCGTCTCTTTTAGTGTTGAAGAAATTGCTCCAGAAACATATAGAGCTATTTATCTTTCGTGGTTTTTGTTAGTGGTTGAGGCTAGTCTTTTGGTTTATATTAATTCCATTTCTGAAAGTAATTAGTAAGATTAACGGAAGATTGGTTGCTGACAATTTTGGGACTGTGGATGTTGGAGCTATTTTAAAAGTAGATGGAAGCAAGTCTTACGTAGAACCTCTCTGGATGAATTGCCACAGATTTGGAATATTTTGGTCGGTCACATGTCGCTTGTAGGACCAATAATAGGTAGAGGAAGTGATTCATTAGCGATTTAGCGACTGATATCCTATGTGGAAGGCGTTTGAAGGTGACTATCACGCGAGATCGATTTTGAGAGACTATTCGAGCAATTGGACGACTTCGAGTTGTGCCGTTTTTACAAACGCAGAGAAAAGACTAGGATTCAGAAAAATTTTAGAGGTGGGTACGCCTTGGAAGATTCGCATGAGAAATGACGTCCTCATCAGAGAGGGATAGCAAGTTATTGTCGGCG |
| C2 | TGATTCAACTGTTCTAATTACCGCTCCAAGAGCATTTACAAACATAGGTCGGTCATTTTATTAGCAGAAGTTTTAAGTCACGGACATCCTTGCGGTTTTACGTGGGGGGACCCTTAGTGACATCTGTAATGCTGAGAAGGCTCTCGGAGGATTGTGTACTTCCTGTTCTAGGCTTGGTGGAGCAGTGGTTGGGTTTGAACAAGAGAGACGCTATTTTCCTCTGGGAAGTGGTTTTGCTACATCTGGAACTGGTTCAGCAACTATACGCTGGCATGGCAGAAAAGTCCTAAGGTATTTCAGACGGTCTTAGAACCTGTCCAATGAAGAGCAAGACTTGACAGTCCTGTTAGTGCACCTGTATTTAACGATAAATGGCGTGAACAACGAATAGGAGATGAAGGAGTTTTGAATGCTGTTCTGGAAGGACATGTCTTGATTGTCCGGTGGAGTAGGTCAAGATGGGAACTATTCAAGTACATTGGCGCTCCGTTACTCAGTATATTGGGGAGAACAGACACCCTAGCTATGGGATGAACCTGTGTTTCAAGCTTCCCCTTTAGACTTTCAAAATACCCAGGACACCCGTTCAACGATACGTGATACTTTGATTTCATTTTTTGTTATGGGTGGGGAGATGAGATATTGATCTAGCTACTTGACTAAAATATTTGAACTGCTAGGGAGCGAGGTCACTAAAAGTCATACAGCGAAATCGACTCGTCTACCAAGATAGGGAGAGCAGCATTAGGGGCAGGTTTTGCTTCATATGGGAGCAACGCTGCTTATGTGTATTACTCTCCCCTGTAGCTAATGATAACTTTTGGCCGATTTTTGAGCAGTTGCTCCTCTATTTGTACCAACGGGGCGTGCGTTATGGACTGGCTGATGGTTCTCTAGGTCAACGTATTGGAACTCTTAGAAATTGGGAAAATAAAGGCTATTATTGGGTCAGGATTTATGCACAGTTTGGTGGTTCTATATCCTCTCCTATCAACTATTACTCATTATACTACCTATCATTACAACTCCCTATGTGACACGGGTCTTTTCTTTGAATGATCTAGGGACTTATGGTTATTCTGATGTTCATGGCTATTATGAAGCGTGGAATTTTCTATACTGCAATAGGGCAATTCTTAGCCGGATTCTCTCACCTGTAGAATATGGTATCGTAGCAGTAGGATTCAATGTACAAGTGGTATCATTATAGTGGGGGTGTTATACGGCGAAACTATTTATGTTAGTTTTCCGC |
| C3 | GAGCTCATGTCTAAGTGAAGTGGTCGATGGTGGGTCCTGTTCCGCTATTCCATCAAGATTTAGCTCGTCGTATTGGACTTTGGCAATGCAGATATCGGTACCCAGAAAATTACTCCTCAGCAAGAGGCACAGTTTGCTCCATTACAACCCAGGCCGAATATGACGAATTAACTACCGTCACCCGCCACAGATTCATGAGCAGATCACCCGTCTGTTCCACGCGTTAGTGCGTTAAGTACTCCCCTCCATTACCCATTTGGTATGGGAGTAGGGAGTGTGGGAGAGGTTCACAAACAGCACTTTCTTCAGAGGAGGAATTGGAATGATAATTGCAACACTCAGTTTCGTTTTATTACCAAGCGCATTCTTCTTCGTCCATAACCTTCTCTAGCCTTGTTGTTACTGGAAACTGATCATAATGCGAAATTTGCTCGCCATACTGCACAATTGGTTTAACCGCAGGCAAAGCATTTAATCTTGGCAATTTTGGGGTTACACAGACATCGACACACACGAGTACATCTACCATCACATCTACGATTACATACTCACATCAACGAGGCGGTGTAGCAATGGTTATTTCGTTCTTTGCTAATCAGGCAGACTGGCCTTGTCTATTCAGCCCTTCTGGTCTCATTGTAGCGGGCATTCTTGAGTTCCCAATTTACAATATAATCGCGAAGGAGAACATAACTATGGAGGACAAAAAAATGAACTAACTCAAATAGTAACGTAAGTCAGGCGTATTCTTCACAAGGGAAATACCGCAAATAAACAAGAAAATTACAGTATGTATCTCTTAAGGTTGAATAGAAAGTTTTGAGCAAATAGATGTATCCCAGTTAACACTACCTTTGAAGCTAGACTATTCGGTCGTGGGACATTAAACCAAAAGAGCTACCGAAGTTGAACTGCGAATATTTAAAGTTGAAAGAGGCTACATCAAATAGTTGGCGAGCTCGGTAGGGGTGAGTACTATTGGAGTGCTTTGGAAGTTACGGTGTGATTCAGTAGTAATTAGTATGGGACAGTTGGGATGATTGGTCGTATTAGTAGATGGCGAATTGCGTGGATCGAACTGAAGAACTAATTGAAGAGGTACATTACGCCAGACCGCCATGGCATACAGTGGTTACTTACGAAAGTATAGGCTTTTTGGT |

**Supplementary Table 4**: Assay-set pools for specific target amplification/Pre-Amplification (Pre-Amp)

| **Pool A (n=32)** | **Pool B (n=32)** | **Pool C (n=34)** |
| --- | --- | --- |
| 1 | 3 | 2 |
| 4 | 6A/C | 6C/D |
| 5 | 7B/C, 40 | 9L/N |
| 6A/B/C/D(F/G/H) | 9A/V | 10A |
| 7A/F | 10B | 11B/C |
| 8 | 11A/B/C/D/F/(E) | 15A/F |
| 9A/L/N/V | 12B | 16F, 18F, 28A/F |
| 10C/F | 13 | 17F |
| 11A/D | 15A/B/C/F | 19A |
| 11F | 16A | 21 |
| 12A/B/F, 44, 46 | 34, 37, 17A | 23A |
| 14 | 18B/C/F | 23B |
| 16F | 19F | 24B/F |
| 15B/C | 22A/F | 25A/F, 38 |
| 18A/B/C | 23F | 27 |
| 19B/F | 25A/F | 29 |
| 20 | 33C | 31 |
| 22F | 35B | 32A/F |
| 23A/B/F | 46 | 33A/F, 37 |
| 24A | 47A/F | 33B |
| 28A/F | Pia*B* | 34 |
| 33D | Bex*A* | 35A/C, 42 |
| 17A | IS481 | 36 |
| 35F, 47F | Ptx*S1* | 39 |
| 41F | pIS1001 | 41A |
| *Eco* | *Mca* | 43 |
| Lyt*A* | *Nla* | 45 |
| *Sag* | *Sau* | 48 |
| *Hin*-b | *Spy* | IgA1 |
| hIS1001 | Xisco | Ply |
| *Nme* | 16S | Bex*B* |
| - | *Sor* | *Pji* |
| - | - | *Aba* |
| - | - | *Kpn* |


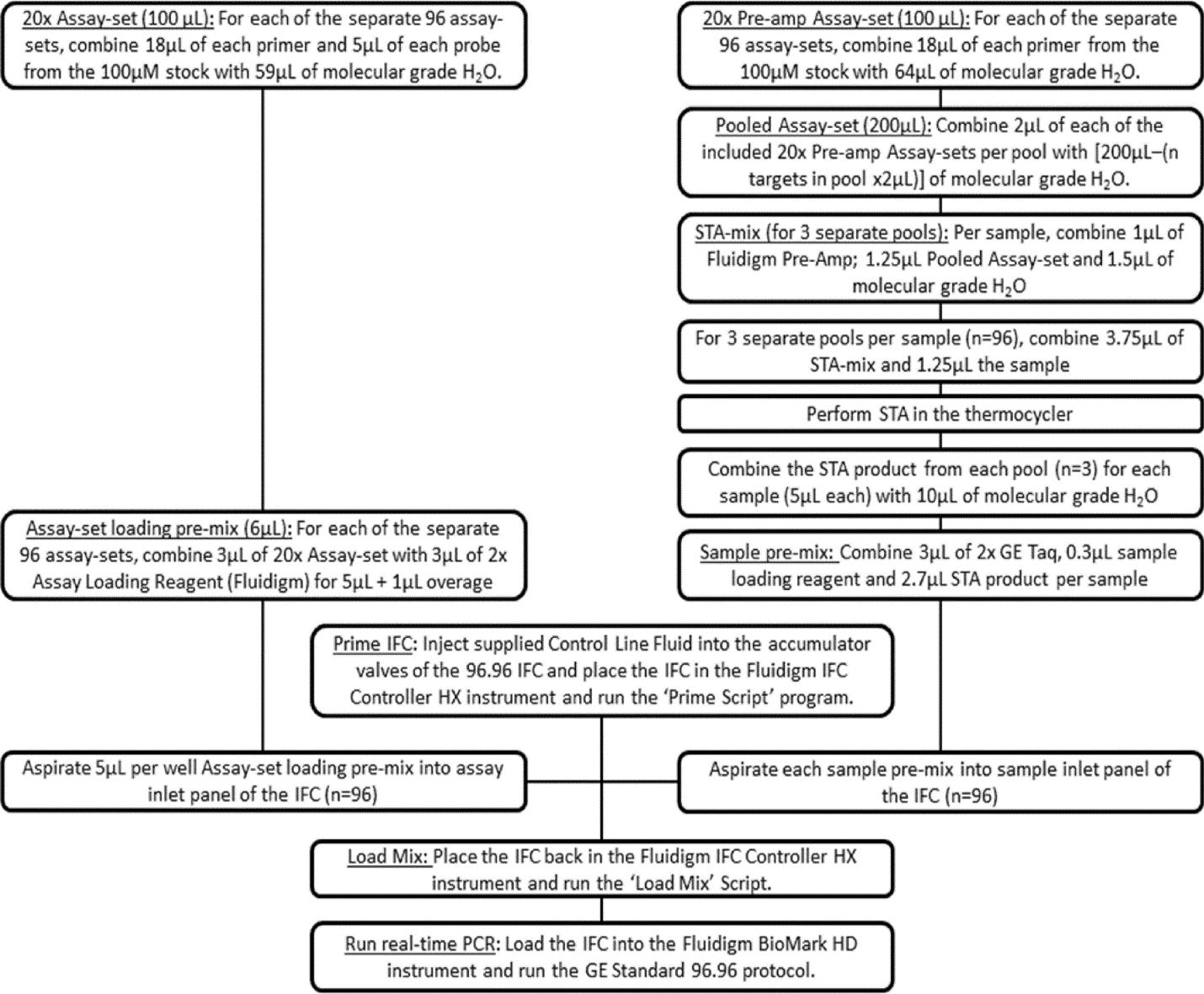


**_Supplementary Figure 1:_** _Flow diagram of the Specific Target Amplification (STA) within single 0.6ml PCR tubes in the Bio-Rad T100 Thermal Cycler and the nano-fluidic high throughput real-time PCR within the 96.96 Gene Expression (GE) Dynamic Array integrated fluidic circuit (IFC) carried out in the IFC Controller HX and the BioMark HD (Standard BioTools ‘Fluidigm’)._

**Supplementary Table 5:** Analytical performance of 5 assay-sets not well optimized in the high-throughput BioMark HD nano-fluidic real-time PCR (‘Fluidigm’)

| **Assay-set** | **Calibrator** | **Efficiency (%)**  **[-1+10^(-1/m)^]** | **LOD**  **(g-Block)** | **Linear equation** | **R^2^** |
| --- | --- | --- | --- | --- | --- |
| Bacterial 16*S* | g-Block | 205 | 10^1^ | y=-2,0649x+23,561 | 0.91 |
| *Streptococcus pneumoniae* (Xisco) | St 4 | 89 | 10^2^ | y=-3.6201x+27.495 | 0.99 |
| *Pneumocystis jiroveci* | g-Block | 91 | 10^2^ | y=-3.5599x+31.266 | 0.94 |
| *Streptococcus algalactiae* (GBS) | GBS | 113 | 10^2^ | y=-3.0364x+24.213 | 0.99 |
| *Escherichia coli* | *E.coli* | 131 | 10^2^ | y=-2.7535x+21.717 | 0.99 |
|  | | | | | |

**Supplementary Table 6:** Geometric Mean Density (GMD) of bacterial and pneumococcal serotype targets quantified using the high-throughput BioMark HD nano-fluidic real-time PCR (‘Fluidigm’ qPCR)

| **Target** | **GMD** **Log_10_ Gene Equivalents (GE)/mL (95%CI)** |
| --- | --- |
| Overall *S. pneumoniae* | 4.2 (4.1-4.2) |
| Sum of serotype density | 4.9 (4.7-5.0) |
| Lyt*A* | 4.4 (4.4-4.5) |
| Pia*B* | 4.2 (4.1-4.2) |
| PCV7-VT | 4.1 (4.0-4.2) |
| PCV13-VT | 4.2 (4.0-4.3) |
| NVT | 3.2 (3.1-3.4) |
| NT | 3.0 (2.9-3.2) |
| 1 | 1.8 (1.3-2.7) |
| 3 | 1.7 (1.4-2.0) |
| 4 | 3.2 (2.9-3.6) |
| 5 | 1.8 (1.7-2.0) |
| 6A | 5.2 (5.0-5.4) |
| 6B | 4.2 (4.1-4.4) |
| 7AF | 2.1 (1.6-2.7) |
| 9AV | 2.9 (2.5-3.4) |
| 14 | 3.3 (3.1-3.6) |
| 18C | 3.1 (2.5-3.8) |
| 19A | 2.6 (2.3-2.8) |
| 19F | 4.0 (3.8-4.2) |
| 23F | 3.8 (3.6-3.9) |
| 2 | 1.7 (0.2-13.0) |
| 6C | 4.7 (3.3-6.7) |
| 6D | 6.3 |
| 7BC40 | 3.9 (3.3-4.5) |
| 8 | 2.5 (1.8-3.5) |
| 9LN | 4.3 (3.8-4.9) |
| 9like | 1.7 (1.5-2.0) |
| 10A | 2.6 (2.0-3.5) |
| 10B | 0.9 (0.7-1.1) |
| 10CF | 2.6 (2.3-3.0) |
| 11AD | 3.9 (3.6-4.2) |
| 11BC | 3.1 (1.2-7.8) |
| 11F | - |
| 12AF44 | 0.9 (0.6-1.3) |
| 12B | - |
| 13 | 2.9 (2.5-3.4) |
| 15AF | 3.5 (3.1-4.0) |
| 15BC | 4.5 (4.3-4.8) |
| 15like | 2.0 (1.9-2.2) |
| 16A | 1.4 (1.0-2.1) |
| 16F | 3.7 (3.4-4.0) |
| 17A | - |
| 17F | 3.3 (2.9-3.8) |
| 18F | - |
| 18A | 0.7 (0.5-0.9) |
| 18B | 2.1 (1.5-2.9) |
| 19B | 0.4 (0.3-0.7) |
| 20 | 2.7 (2.3-3.3) |
| 21 | 4.2 (3.4-5.0) |
| 22A | 1.8 (1.7-1.9) |
| 22F | 5.8 (4.7-7.1) |
| 23A | 3.3 (3.0-3.7) |
| 23B | 4.1 (3.9-4.4) |
| 24A | 2.5 (1.7-3.7) |
| 24BF | 1.4 |
| 25AF | - |
| 27 | 1.8 (1.0-3.2) |
| 28AF | - |
| 29 | 2.1 (1.8-2.4) |
| 31 | 1.9 (1.3-2.8) |
| 32AF | 1.1 (0.6-2.2) |
| 33AF | 1.2 (0.8-1.7) |
| 33B | 1.9 (1.6-2.2) |
| 33C | 1.9 (1.2-3.1) |
| 33D | - |
| 34 | 3.4 (3.1-3.7) |
| 35AC42 | 2.9 (2.4-3.4) |
| 35B | 3.1 (2.6-3.7) |
| 35F | 2.0 (1.5-2.5) |
| 36 | 1.9 (1.3-2.9) |
| 37 | - |
| 38 | - |
| 39 | 0.5 (0.4-0.7) |
| 41A | 1.6 (0.6-4.7) |
| 41F | 0.6 (0.4-0.9) |
| 43 | 2.8 (2.3-3.4) |
| 45 | 1.7 (1.5-2.0) |
| 46 | 3.1 (2.6-3.7) |
| 47F | 1.6 (0.3-8.0) |
| 47A | 1.2 (1.0-1.3) |
| 48 | 1.4 (0.9-2.1) |
| *A. baumannii* | 1.9 (1.7-2.0) |
| *K. pneumoniae* | 1.1 (0.9-1.4) |
| *M. catarrhalis* | 4.1 (4.0-4.1) |
| *N. lactamica* | 2.2 (2.1-2.4) |
| *N. meningitidis* | 1.2 (0.9-1.5) |
| *S. aureus* | 3.0 (2.9-3.2) |
| *S. oralis* | 2.5 (2.5-2.6) |
| *S. pyogenes* | 1.6 (1.4-1.9) |
| *H. influenzae type b* | 4.5 (4.1-5.0) |
| *H. influenzae (non-type b)* | 4.6 (4.0-5.3) |
| *NTHI* | 2.5 (2.4-2.6) |
| *B. pertussis* | - |
| *B. holmesii* | 5.2 (4.1-6.4) |
| *B. parapertussis/bronchiseptica* | 1.5 (0.5-4.6) |

**_
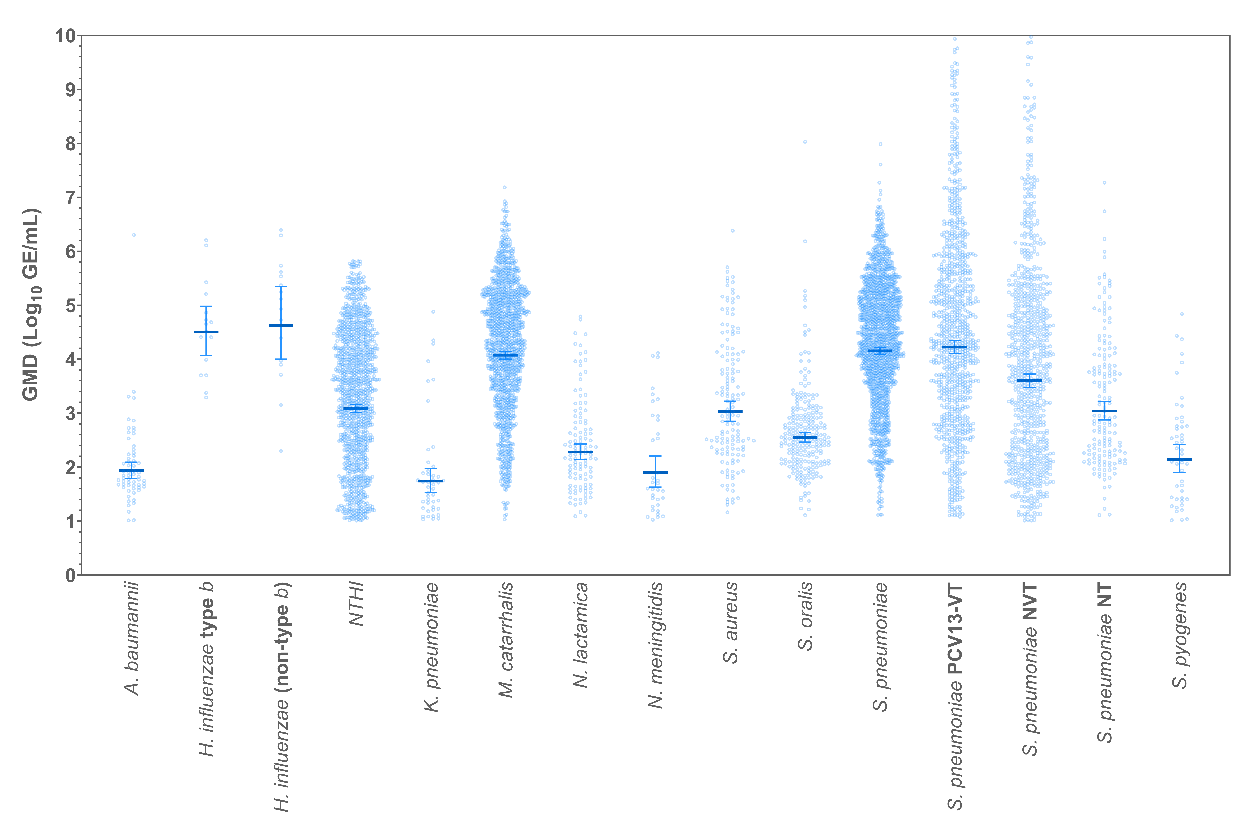
_**

**_Supplementary Figure 2: Geometric Mean Density (GMD) measured in Log10 Gene Equivalents (GE)/mL._** _GMD was determined using ‘Fluidigm’ qPCR. Error Bars represent 95% Confidence intervals. PCv13VT include vaccine serotypes (1, 3, 4, 5,6A, 6B, 7A/F, 9A/V, 14, 18C, 19A, 19F and 23F); NVT, all serotypes/serogroups not included in PCV13. NT, non-typeable S. pneumoniae._

**Supplementary Table 7**: Hierarchy of co-colonising pneumococcal serotypes detected in Nasopharyngeal Swab samples collected from Rural (Agincourt, 2009) and Urban (Soweto, 2010) children 0-60 months-of-age.

| **Serogroup/type** | **Primary Coloniser % (n/N) ^†^** | **Co-Colonisation  % (n/N) ^†^** | **Second Serotype % (n/N) ^†^** | **Third Serotype % (n/N) ^†^** | **≥ Four Serotypes % (n/N) ^†^** |
| --- | --- | --- | --- | --- | --- |
| PCV7 VT | 42.6 (302/709) | **57.4 (407/709)** | 47.4 (336/709) | 8.5 (60/709) | 1.6 (11/709) |
| Additional PCV13 VT | 26.7 (96/359) | **73.3 (263/359)** | 42.9 (154/359) | 23.1 (83/359) | 7.2 (26/359) |
| PCV13 VT | 43.8 (425/971) | **56.2 (546/971)** | 48.6 (472/971) | 7.6 (74/971) |  |
| NVT | **67.1 (621/926)** | 32.9 (305/926) | 10.0 (93/926) | 20.8 (193/926) | 2.1 (19/926) |
| NT | **50.6 (85/168)** | 49.4 (83/168) | 49.4 (83/168) |  |  |
| 1 | 23.1 (3/13) | **76.9 (10/13)** | 30.8 (4/13) | 46.2 (6/13) |  |
| 3 | 33.3 (17/51) | **66.7 (34/51)** | 47.1 (24/51) | 15.7 (8/51) | 3.9 (2/51) |
| 4 | 51.7 (15/29) | 48.3 (14/29) | 37.9 (11/29) | 10.3 (3/29) |  |
| 5 | 23.2 (13/56) | **76.8 (43/56)** | 42.9 (24/56) | 17.9 (10/56) | 16.1 (9/56) |
| 6A | **93.7 (133/142)** | 6.3 (9/142) | 6.3 (9/142) |  |  |
| 6B | **80.6 (158/196)** | 19.4 (38/196) | 16.3 (32/196) | 3.1 (6/196) |  |
| 7A/F | 33.3 (1/3) | **66.7 (2/3)** | 66.7 (2/3) | 0.0 (/3) |  |
| 9A/V | 56.3 (18/32) | 43.8 (14/32) | 34.4 (11/32) | 9.4 (3/32) |  |
| 14 | 62.1 (59/95) | 37.9 (36/95) | 32.6 (31/95) | 3.2 (3/95) | 2.1 (2/95) |
| 18C | 88.2 (15/17) | 11.8 (2/17) | 11.8 (2/17) |  |  |
| 19A | 60.2 (71/118) | 39.8 (47/118) | 23.7 (28/118) | 8.5 (10/118) | 7.6 (9/118) |
| 19F | 78.3 (191/244) | 21.7 (53/244) | 17.2 (42/244) | 3.7 (9/244) | 0.8 (2/244) |
| 23F | 81.9 (136/166) | 18.1 (30/166) | 13.3 (22/166) | 4.2 (7/166) | 0.6 (1/166) |
| 2 |  | 100.0 (2/2) | 50.0 (1/2) |  | 50.0 (1/2) |
| 6C | 100.0 (5/5) |  |  |  |  |
| 6D | 100.0 (1/1) |  |  |  |  |
| 7B/C/40 | 70.0 (14/20) | 30.0 (6/20) | 25.0 (5/20) | 5.0 (1/20) |  |
| 8 | 46.2 (6/13) | 53.8 (7/13) | 46.2 (6/13) | 7.7 (1/13) |  |
| 9-Like | 26.1 (6/23) | 73.9 (17/23) | 30.4 (7/23) | 21.7 (5/23) | 21.7 (5/23) |
| 9L/N | 71.4 (10/14) | 28.6 (4/14) | 21.4 (3/14) | 7.1 (1/14) |  |
| 10A | 75.0 (15/20) | 25.0 (5/20) | 10.0 (2/20) | 10.0 (2/20) | 5.0 (1/20) |
| 10B | 5.3 (1/19) | 94.7 (18/19) | 26.3 (5/19) | 31.6 (6/19) | 36.8 (7/19) |
| 10C/F | 10.0 (1/10) | 90.0 (9/10) | 70.0 (7/10) | 20.0 (2/10) |  |
| 11A/D | 70.0 (35/50) | 30.0 (15/50) | 28.0 (14/50) | 2.0 (1/50) |  |
| 11B/C | 66.7 (2/3) | 33.3 (1/3) | 33.3 (1/3) |  |  |
| 12A/F/44 | 23.5 (4/17) | 76.5 (13/17) | 17.6 (3/17) | 52.9 (9/17) | 5.9 (1/17) |
| 13 | 65.9 (27/41) | 34.1 (14/41) | 29.3 (12/41) | 4.9 (2/41) |  |
| 15A/F | 55.0 (11/20) | 45.0 (9/20) | 45.0 (9/20) |  |  |
| 15B/C | 94.3 (66/70) | 5.7 (4/70) | 5.7 (4/70) |  |  |
| 15-Like | 33.1 (40/121) | 66.9 (81/121) | 37.2 (45/121) | 19.8 (24/121) | 9.9 (12/121) |
| 16A |  | 100.0 (5/5) | 20.0 (1/5) | 20.0 (1/5) | 60.0 (3/5) |
| 16F | 79.7 (47/59) | 20.3 (12/59) | 18.6 (11/59) | 1.7 (1/59) |  |
| 17F | 61.4 (27/44) | 38.6 (17/44) | 29.5 (13/44) | 6.8 (3/44) | 2.3 (1/44) |
| 18A | 15.4 (6/39) | 84.6 (33/39) | 46.2 (18/39) | 30.8 (12/39) | 7.7 (3/39) |
| 18B | 37.5 (6/16) | 62.5 (10/16) | 43.8 (7/16) | 12.5 (2/16) | 6.3 (1/16) |
| 19B | 14.6 (7/48) | 85.4 (41/48) | 45.8 (22/48) | 16.7 (8/48) | 22.9 (11/48) |
| 20 | 79.4 (27/34) | 20.6 (7/34) | 11.8 (4/34) | 8.8 (3/34) |  |
| 21 | 80.0 (12/15) | 20.0 (3/15) | 20.0 (3/15) |  |  |
| 22A | 19.0 (16/84) | 81.0 (68/84) | 42.9 (36/84) | 31.0 (26/84) | 7.1 (6/84) |
| 22F | 100.0 (6/6) |  |  |  |  |
| 23A | 52.9 (27/51) | 47.1 (24/51) | 45.1 (23/51) | 2.0 (1/51) |  |
| 23B | 84.0 (42/50) | 16.0 (8/50) | 16.0 (8/50) |  |  |
| 24A | 33.3 (1/3) | 66.7 (2/3) |  | 66.7 (2/3) |  |
| 24B/F |  | 100.0 (1/1) |  |  | 100.0 (1/1) |
| 27 | 28.6 (2/7) | 71.4 (5/7) | 14.3 (1/7) | 28.6 (2/7) | 28.6 (2/7) |
| 29 |  | 100.0 (8/8) | 75.0 (6/8) | 12.5 (1/8) | 12.5 (1/8) |
| 31 | 44.4 (8/18) | 55.6 (10/18) | 16.7 (3/18) | 27.8 (5/18) | 11.1 (2/18) |
| 32A/F | 16.7 (1/6) | 83.3 (5/6) | 0.0 (/6) | 50.0 (3/6) | 33.3 (2/6) |
| 33A/F | 28.6 (4/14) | 71.4 (10/14) | 21.4 (3/14) | 14.3 (2/14) | 35.7 (5/14) |
| 33B |  | 100.0 (5/5) | 60.0 (3/5) | 20.0 (1/5) | 20.0 (1/5) |
| 33C | 12.5 (1/8) | 87.5 (7/8) | 25.0 (2/8) | 12.5 (1/8) | 50.0 (4/8) |
| 34 | 75.6 (34/45) | 24.4 (11/45) | 20.0 (9/45) | 4.4 (2/45) |  |
| 35A/C/42 | 62.5 (20/32) | 37.5 (12/32) | 18.8 (6/32) | 6.3 (2/32) | 12.5 (4/32) |
| 35B | 60.0 (15/25) | 40.0 (10/25) | 24.0 (6/25) | 12.0 (3/25) | 4.0 (1/25) |
| 35F | 25.0 (4/16) | 75.0 (12/16) | 37.5 (6/16) | 25.0 (4/16) | 12.5 (2/16) |
| 36 |  | 100.0 (3/3) | 33.3 (1/3) | 66.7 (2/3) |  |
| 39 | 11.1 (1/9) | 88.9 (8/9) | 22.2 (2/9) | 44.4 (4/9) | 22.2 (2/9) |
| 41A | 20.0 (1/5) | 80.0 (4/5) | 20.0 (1/5) | 20.0 (1/5) | 40.0 (2/5) |
| 41F | 8.3 (1/12) | 91.7 (11/12) | 16.7 (2/12) | 33.3 (4/12) | 41.7 (5/12) |
| 43 | 27.3 (3/11) | 72.7 (8/11) | 54.5 (6/11) | 9.1 (1/11) | 9.1 (1/11) |
| 45 | 10.0 (2/20) | 90.0 (18/20) | 65.0 (13/20) | 15.0 (3/20) | 10.0 (2/20) |
| 46 | 66.7 (4/6) | 33.3 (2/6) | 16.7 (1/6) |  | 16.7 (1/6) |
| 47A | 10.3 (3/29) | 89.7 (26/29) | 48.3 (14/29) | 24.1 (7/29) | 17.2 (5/29) |
| 47F |  | 100.0 (2/2) | 50.0 (1/2) |  | 50.0 (1/2) |
| 48 | 20.0 (1/5) | 80.0 (4/5) | 20.0 (1/5) |  | 60.0 (3/5) |
| Total | **59.7 (1403/2352)** | 40.3 (949/2352) | 25.5 (599/2352) | 9.6 (226/2352) | 5.3 (124/2352) |
| **^†^**n is the number of isolates in each category for each period and N is the total isolates identified as each serotype/group. The rank was determined according to colonization density. | | | | | |


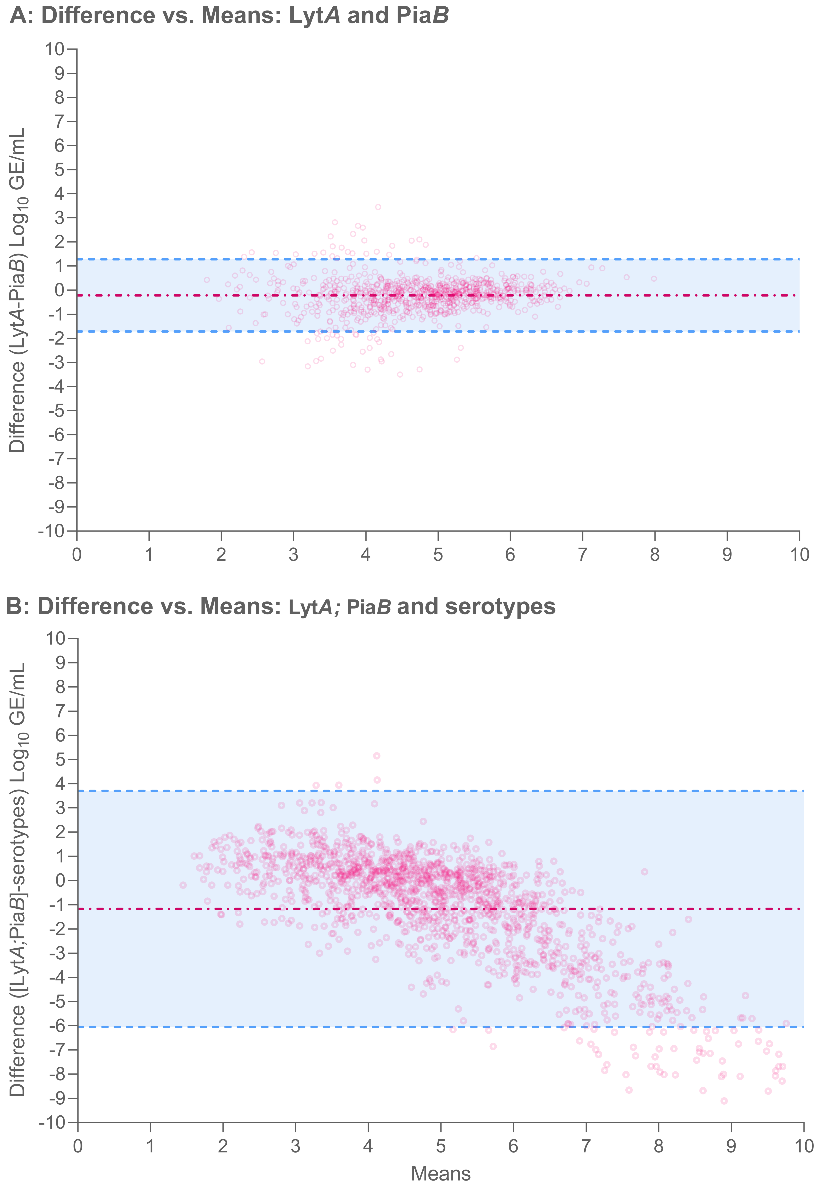


**_Supplementary Figure 3: Concordance of pneumococcal quantification by LytA, PiaB and the sum of all serotype-specific assay-sets (Bland-Altman plots depicting the difference vs means)._** _Panel A compares pneumococcal density determined by the Lyt_*_A_* _and Pia_*_B_* _assay-sets in ‘Fluidigm’ qPCR. Panel B compares average pneumococcal density (Lyt_*_A_* _and Pia_*_B_*_) to the total density of all serotype-specific assay-sets in ‘Fluidigm’ qPCR. The light blue shaded region indicates the 95% limits of agreement and the dark pink dotted line represents the line of bias (mean difference). The y-axis on both graphs is limited to the range of -10 to 10 Log10 Gene Equivalents (GE)/mL._

References:

[1] C. Azzari, M. Moriondo, G. Indolfi, M. Cortimiglia, C. Canessa, L. Becciolini, F. Lippi, M. de Martino, and M. Resti, Realtime PCR is more sensitive than multiplex PCR for diagnosis and serotyping in children with culture negative pneumococcal invasive disease. PLoS One 5 (2010) e9282.

[2] S. Pholwat, F. Sakai, P. Turner, J.E. Vidal, and E.R. Houpt, Development of a TaqMan Array Card for Pneumococcal Serotyping on Isolates and Nasopharyngeal Samples. J. Clin. Microbiol. 54 (2016) 1842-50.

[3] S.L. Downs, S.A. Madhi, L. Van der Merwe, M.C. Nunes, and C.P. Olwagen, High-throughput nanofluidic real-time PCR to discriminate Pneumococcal Conjugate Vaccine (PCV)-associated serogroups 6, 18, and 22 to serotypes using modified oligonucleotides. Scientific Reports 11 (2021) 23728.

[4] C.P. Olwagen, P.V. Adrian, and S.A. Madhi, Comparison of traditional culture and molecular qPCR for detection of simultaneous carriage of multiple pneumococcal serotypes in African children. Sci. Rep. 7 (2017) 4628.

[5] F. Sakai, G. Sonaty, D. Watson, K.P. Klugman, and J.E. Vidal, Development and characterization of a synthetic DNA, NUversa, to be used as a standard in quantitative polymerase chain reactions for molecular pneumococcal serotyping. FEMS Microbiol. Lett. 364 (2017).

[6] M. Messaoudi, M. Milenkov, W.C. Albrich, M.P.G. van der Linden, T. Bénet, M. Chou, M. Sylla, P. Barreto Costa, N. Richard, K.P. Klugman, H.P. Endtz, G. Paranhos-Baccalà, and J.-N. Telles, The Relevance of a Novel Quantitative Assay to Detect up to 40 Major Streptococcus pneumoniae Serotypes Directly in Clinical Nasopharyngeal and Blood Specimens. PLOS ONE 11 (2016) e0151428.

[7] F. Sakai, S. Chochua, C. Satzke, E.M. Dunne, K. Mulholland, K.P. Klugman, and J.E. Vidal, Single-plex quantitative assays for the detection and quantification of most pneumococcal serotypes. PLoS One 10 (2015) e0121064.

[8] N.J. Gadsby, M.P. McHugh, C.D. Russell, H. Mark, A. Conway Morris, I.F. Laurenson, A.T. Hill, and K.E. Templeton, Development of two real-time multiplex PCR assays for the detection and quantification of eight key bacterial pathogens in lower respiratory tract infections. Clinical Microbiology and Infection 21 (2015) 788.e1-788.e13.

[9] K.M. Tatti, K.N. Sparks, K.O. Boney, and M.L. Tondella, Novel Multitarget Real-Time PCR Assay for Rapid Detection of &lt;span class=&quot;named-content genus-species&quot; id=&quot;named-content-1&quot;&gt;Bordetella&lt;/span&gt; Species in Clinical Specimens. Journal of Clinical Microbiology 49 (2011) 4059.

[10] D.-Y. Lee, K. Shannon, and L.A. Beaudette, Detection of bacterial pathogens in municipal wastewater using an oligonucleotide microarray and real-time quantitative PCR. Journal of Microbiological Methods 65 (2006) 453-467.

[11] Y. Maaroufi, J.-M. De Bruyne, C. Heymans, and F. Crokaert, Real-Time PCR for Determining Capsular Serotypes of Haemophilus influenzae. Journal of Clinical Microbiology 45 (2007) 2305.

[12] J. Dolan Thomas, C.P. Hatcher, D.A. Satterfield, M.J. Theodore, M.C. Bach, K.B. Linscott, X. Zhao, X. Wang, R. Mair, S. Schmink, K.E. Arnold, D.S. Stephens, L.H. Harrison, R.A. Hollick, A.L. Andrade, J. Lamaro-Cardoso, A.P.S. de Lemos, J. Gritzfeld, S. Gordon, A. Soysal, M. Bakir, D. Sharma, S. Jain, S.W. Satola, N.E. Messonnier, and L.W. Mayer, sodC-Based Real-Time PCR for Detection of Neisseria meningitidis. PLOS ONE 6 (2011) e19361.

[13] L. Jensen, A.V. Jensen, G. Praygod, J. Kidola, D. Faurholt-Jepsen, J. Changalucha, N. Range, H. Friis, J. Helweg-Larsen, J.S. Jensen, and A.B. Andersen, Infrequent detection of Pneumocystis jirovecii by PCR in oral wash specimens from TB patients with or without HIV and healthy contacts in Tanzania. BMC Infectious Diseases 10 (2010) 140.

[14] J.C. McAvin, P.A. Reilly, R.M. Roudabush, W.J. Barnes, A. Salmen, G.W. Jackson, K.K. Beninga, A. Astorga, F.K. McCleskey, W.B. Huff, D. Niemeyer, and K.L. Lohman, Sensitive and specific method for rapid identification of Streptococcus pneumoniae using real-time fluorescence PCR. J Clin Microbiol 39 (2001) 3446-51.

[15] O. Greiner, P.J.R. Day, P.P. Bosshard, F. Imeri, M. Altwegg, and D. Nadal, Quantitative Detection of Streptococcus pneumoniae in Nasopharyngeal Secretions by Real-Time PCR. Journal of Clinical Microbiology 39 (2001) 3129-3134.

[16] K. Trzciński, D. Bogaert, A. Wyllie, M.L.J.N. Chu, A. van der Ende, J.P. Bruin, G. van den Dobbelsteen, R.H. Veenhoven, and E.A.M. Sanders, Superiority of Trans-Oral over Trans-Nasal Sampling in Detecting Streptococcus pneumoniae Colonization in Adults. PLOS ONE 8 (2013) e60520.

[17] M. Kodani, G. Yang, L.M. Conklin, T.C. Travis, C.G. Whitney, L.J. Anderson, S.J. Schrag, T.H. Taylor, B.W. Beall, R.F. Breiman, D.R. Feikin, M.K. Njenga, L.W. Mayer, M.S. Oberste, M.L.C. Tondella, J.M. Winchell, S.L. Lindstrom, D.D. Erdman, and B.S. Fields, Application of TaqMan Low-Density Arrays for Simultaneous Detection of Multiple Respiratory Pathogens. Journal of Clinical Microbiology 49 (2011) 2175.

[18] CDC, Centers for Disease Control and Prevention, Streptococcus Laboratory Protocols. Accessed 30-Jan-2016.

[19] G. Àlvarez, M. González, S. Isabal, V. Blanc, and R. León, Method to quantify live and dead cells in multi-species oral biofilm by real-time PCR with propidium monoazide. AMB Express 3 (2013) 1.

[20] M.A. Nadkarni, F.E. Martin, N.A. Jacques, and N. Hunter, Determination of bacterial load by real-time PCR using a broad-range (universal) probe and primers set. Microbiology 148 (2002) 257-266.
